# Radiotherapy transiently reduces the sensitivity of cancer cells to lymphocyte cytotoxicity

**DOI:** 10.1073/pnas.2111900119

**Published:** 2022-01-18

**Authors:** Karoliina Tuomela, Debayan Mukherjee, Ashley R. Ambrose, Ashish Harikrishnan, Holly Mole, Adam Hurlstone, Björn Önfelt, Jamie Honeychurch, Daniel M. Davis

**Affiliations:** ^a^The Lydia Becker Institute of Immunology and Inflammation, The University of Manchester, Manchester M13 9NT, United Kingdom;; ^b^Division of Cancer Sciences, The University of Manchester, Manchester M13 9PL, United Kingdom;; ^c^Department of Microbiology, Tumor and Cell Biology, Karolinska Institutet, 17177 Stockholm, Sweden;; ^d^Department of Applied Physics, Science for Life Laboratory, Kungliga Tekniska Högskolan Royal Institute of Technology, 17165 Stockholm, Sweden

**Keywords:** NK cell, T cell, perforin, cancer, radiotherapy

## Abstract

The impact of radiotherapy on the interaction between immune cells and cancer cells is important not least because radiotherapy can be used alongside immunotherapy as a cancer treatment. Unexpectedly, we found that X-ray irradiation of cancer cells induced significant resistance to natural killer (NK) cell killing. This was true across a wide variety of cancer-cell types as well as for antibody-dependent cellular cytotoxicity. Resistance appeared 72 h postirradiation and persisted for 2 wk. Resistance could also occur independently of radiotherapy through pharmacologically induced cell-cycle arrest. Crucially, multiple steps in NK-cell engagement, synapse assembly, and activation were unaffected by target cell irradiation. Instead, radiotherapy caused profound resistance to perforin-induced calcium flux and lysis. Resistance also occurred to a structurally similar bacterial toxin, streptolysin O. Radiotherapy did not affect the binding of pore-forming proteins at the cell surface or membrane repair. Rather, irradiation instigated a defect in functional pore formation, consistent with phosphatidylserine-mediated perforin inhibition. In vivo, radiotherapy also led to a significant reduction in NK cell–mediated clearance of cancer cells. Radiotherapy-induced resistance to perforin also constrained chimeric antigen receptor T-cell cytotoxicity. Together, these data establish a treatment-induced resistance to lymphocyte cytotoxicity that is important to consider in the design of radiotherapy–immunotherapy protocols.

## Significance

Radiotherapy is one of the most common forms of cancer treatment used today. Its impact in directly killing cancer cells, and on antitumour immunity, is well recognized, but its effect on immune cell–cancer cell interactions is not fully understood. Here, we show that irradiation of cancer cells surprisingly leads to resistance against immune cell cytotoxicity. This was mediated by a reduced susceptibility to the pore-forming protein perforin. Resistance manifested as a reduction in both necrotic death following osmotic lysis and apoptotic death following granzyme B uptake. Overall, these data establish a form of treatment-induced resistance to lymphocyte cytotoxicity. These findings have significant implications for the optimal design of radiotherapy–immunotherapy protocols.

Radiotherapy is one of the most commonly used treatments for solid tumors, with approximately half of patients receiving radiotherapy as part of their treatment (1). The classical model for radiotherapy-induced tumor regression involves the production of DNA damage leading to cell-cycle arrest and cell death (2). However, it is now widely recognized that radiotherapy also alters the antitumor immune response in a manner that can critically affect the outcome of treatment (3). Understanding how irradiation affects antitumor immunity by T cells, dendritic cells, and macrophages has driven the development of new immunotherapies that enhance the efficacy of radiotherapy (4). In contrast, little is known about how radiotherapy affects the interaction between natural killer (NK) cells and cancer cells.

NK cells, like cytotoxic T cells, contribute to tumor control by secreting cytotoxic granules containing perforin and granzyme B or by expressing ligands, such as TNF-related apoptosis-inducing ligand (TRAIL) and Fas ligand, that bind death receptors on target cells (5–7). Secretion of these cytotoxic effector proteins occurs following formation of a lytic immune synapse (8, 9). NK cells polarize the microtubule organizing center (MTOC) and cytotoxic granules toward the immune synapse and then release granule contents into the synaptic cleft in order to kill the target cell (10). Subsequent detachment permits NK cells to carry out multiple sequential kills (11, 12). Novel immunotherapies that harness the effector functions of NK cells have recently been developed for the treatment of cancer (13). However, more must be understood about how radiotherapy affects NK cell–cancer cell interactions if these treatments are to be used effectively in combination.

Cancer radiotherapy has been reported to modulate a number of factors which, at least in principle, could influence NK-cell activity. In particular, radiotherapy could up-regulate the expression of stress-inducible activating ligands on cancer cells, which would be expected to increase NK cell–mediated killing (14–18). However, the dynamics of the interaction between NK cells and irradiated cells have not been well studied. Therefore, we set out to test the effect of cancer-cell irradiation on the ability of NK cells to interact with and kill cancer cells. We found that, contrary to what was expected, irradiation of cancer cells resulted in a reduced susceptibility to NK-cell killing. We determined that this was caused by an increased resistance to perforin. This also led to reduced killing by cytotoxic T cells. Thus, these data have broad implications for cancer therapies, especially for combinations of radiotherapy and immunotherapy.

## Results

### Irradiated Cancer Cells Become Resistant to NK-Cell Cytotoxicity.

**Fig. 1.**
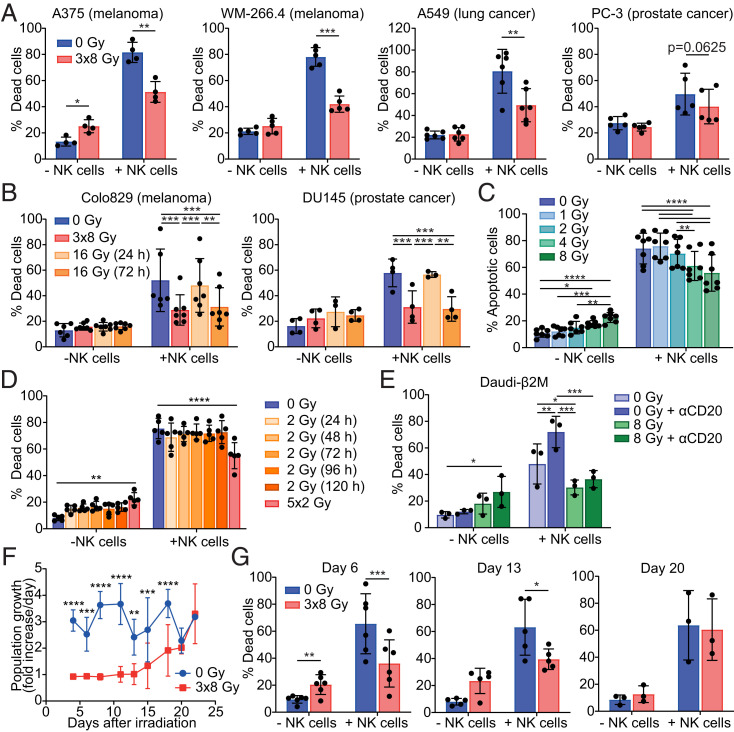
Cancer cells treated with radiotherapy become resistant to NK-cell cytotoxicity. (*A* and *B*) Cancer cell lines were treated with 3 × 8 Gy or a single 16 Gy dose 24 or 72 h prior to assessing cell death in the presence or absence of NK cells over 5 h (A375 [*n* = 4], WM-266.4 [*n* = 5], A549 [*n* = 6], PC-3 [*n* = 5], Colo829 [*n* = 7], and DU145 [*n* = 4]). Death was quantified by annexin V/PI staining using flow cytometry. (*C*) Colo829 cells were treated with single doses of radiation (1 to 8 Gy) 72 h prior to incubation with NK cells as in *A* (*n* = 3). (*D*) Colo829 cells were treated with either five doses of 2 Gy given on consecutive days or a single 2 Gy dose given 24 to 120 h prior to incubation with NK cells as in *A* (*n* = 5). (*E*) Daudi-β2M cells were irradiated with 8 Gy 72 h prior, opsonized with 10 µg/mL anti-CD20 or an isotype-matched control for 30 min, and incubated with or without NK cells for 2 h. Death was determined as in *A* (*n* = 3). (*F* and *G*) Untreated or 3 × 8 Gy–treated Colo829 cells were cultured for 22 d after treatment. Fold change per day was measured at 2- to 3-d intervals (*F*). Cells were harvested at days 6, 13, and 20, and a killing assay with NK cells was carried out (*n* = 3 to 6) (*G*). Means ± SD are indicated. Significance was determined using a paired *t* test (*A* and *G*), one-way ANOVA (*B*–*E*), or two-way ANOVA (*F*). *P < 0.05, **P < 0.01, ***P < 0.001, and ****P < 0.0001.

To investigate the effect of irradiation on the susceptibility of cancer cells to NK-cell cytotoxicity, the response of peripheral blood human NK cells was measured against cell lines derived from different solid tumors: melanoma (Colo829, A375, and WM-266.4), prostate cancer (DU145 and PC-3), and lung cancer (A549). We initially treated cancer cells with a hypofractionated dose of radiation, consisting of a small number of high doses, as this form of radiotherapy has been previously shown to work well in combination with immunotherapies during cancer treatment (19, 20). Unexpectedly, cells treated with three daily fractions of 8 Gy (3 × 8 Gy) were killed significantly less by NK cells compared to mock irradiated (0 Gy) cells (Fig. 1 *A* and *B*). This reduction in killing occurred for all six cell lines tested, with reductions between 39 and 77%. To determine the effect of radiation dose fractionation and timing on resistance to NK-cell cytotoxicity, the fractionated 3 × 8 Gy dose was compared to a single biologically equivalent dose of 16 Gy given either 72 or 24 h prior to incubation with NK cells (Fig. 1*B*). Single-dose irradiation given 72 h prior to incubation with NK cells induced an equivalent reduction in the killing of both Colo829 (61 ± 22%) and DU145 (85 ± 19%) cells compared to 3 × 8 Gy. In contrast, cancer-cell killing was unaffected by 16 Gy given 24 h prior. This indicates that there is a time lag before radiation induces resistance to NK-cell cytotoxicity.

We also explored the impact of lower radiation doses delivered as a single or fractionated regimen. The minimum single dose required to induce resistance to NK-cell killing in Colo829 cells was found to be 4 Gy (Fig. 1*C*). Importantly, fractionated radiotherapy giving five daily doses of 2 Gy led to a significant reduction in susceptibility to NK-cell killing (56 ± 18%; Fig. 1*D*). This indicates that cumulative low doses also induce resistance.

NK cell–mediated antibody-dependent cellular cytotoxicity (ADCC) is a crucial functional pathway utilized by multiple monoclonal antibody immunotherapies such as rituximab, cetuximab, and trastuzumab (21). To determine whether ADCC is also affected by irradiation, a B-cell lymphoma line (Daudi-β2M) transfected to express β2-microglobulin to increase expression of inhibitory class I HLA (22) was treated with a single dose of 8 Gy, cultured for 72 h, opsonized with an anti-CD20 antibody, rituximab, and then incubated with NK cells. Opsonization of nonirradiated Daudi-β2M cells significantly increased NK-cell killing (Fig. 1*E*). However, when irradiated cells were opsonized with rituximab, killing remained significantly lower compared to nonirradiated cells. Thus, irradiation also induced resistance against NK cell–mediated ADCC.

To understand the long-term effect of irradiation on the susceptibility of cancer cells to NK-cell cytotoxicity, 3 × 8 Gy–treated Colo829 cells were cultured for ∼3 wk and their susceptibility to NK-cell lysis was tested at days 6, 13, and 20 (Fig. 1 *F* and *G*). Irradiated Colo829 cells remained in population growth stasis for ∼15 d posttreatment, but population growth recovered by day 20 (Fig. 1*F*). Importantly, Colo829 cells retained their resistance to NK-cell cytotoxicity at 6 d and 13 d posttreatment, the period during which cell proliferation was halted (Fig. 1*G*). However, following resumption of normal growth by day 20, irradiated cells were again killed by NK cells similarly to nonirradiated cells. Altogether, this establishes a time window during which irradiated cells are resistant to NK cell–mediated attack.

### Prolonged Cell-Cycle Arrest Leads to Resistance to NK-Cell Cytotoxicity.

Because nonproliferating irradiated cells were resistant to NK-cell cytotoxicity, we next explored a link between the cell cycle and resistance. After a single dose of 16 Gy, Colo829 cells were arrested in the G2/M phase within 24 h posttreatment, with further accumulation at this phase occurring over time (*SI Appendix*, Fig. 1*A*).

G2/M arrest can also be induced pharmacologically using nocodazole, a microtubule polymerization inhibitor (23). Treatment of Colo829 cells with nocodazole induced G2/M arrest within 24 h, which progressed to polyploidy at 72 h posttreatment (*SI Appendix*, Fig. 1*B*), indicating failed cytokinesis and continued arrest (24). Notably, as with irradiated cells, NK-cell cytotoxicity was significantly reduced after 72 h but not 24 h posttreatment (*SI Appendix*, Fig. 1*C*).

Next, Colo829 cells were treated with palbociclib, a cyclin-dependent kinase 4/6 inhibitor that arrests cells at G0/1 (25) (*SI Appendix*, Fig. 1*D*). Despite palbociclib-treated cells having a very similar distribution within the cell cycle to untreated cells, they were significantly more resistant to NK-cell cytotoxicity after 72 h (*SI Appendix*, Fig. 1*E*).

To test whether or not these effects are due to the DNA damage response, DNA double-strand breaks were visualized by confocal microscopy of phosphorylated histone H2AX (γ-H2AX), a marker of this response. Treatment with nocodazole led to a time-dependent accumulation of γ-H2AX foci, whereas palbociclib did not induce detectable levels of DNA damage (*SI Appendix*, Fig. 1 *H* and *I*). Thus, prolonged cell-cycle arrest, triggered independently of radiotherapy or DNA damage, by pharmaceutical compounds can also lead to resistance to NK-cell cytotoxicity.

### NK-Cell Interactions with Cancer Cells Are Not Affected by Irradiation.

To search for the mechanism behind the impairment in NK-cell cytotoxicity, we next tested the effect of target cell irradiation on four crucial steps required for efficient NK-cell cytotoxicity: conjugation to the target cell, polarization of the MTOC, release of lytic granules (degranulation), and detachment from the target cell (which is important for serial killing).

**Fig. 2.**
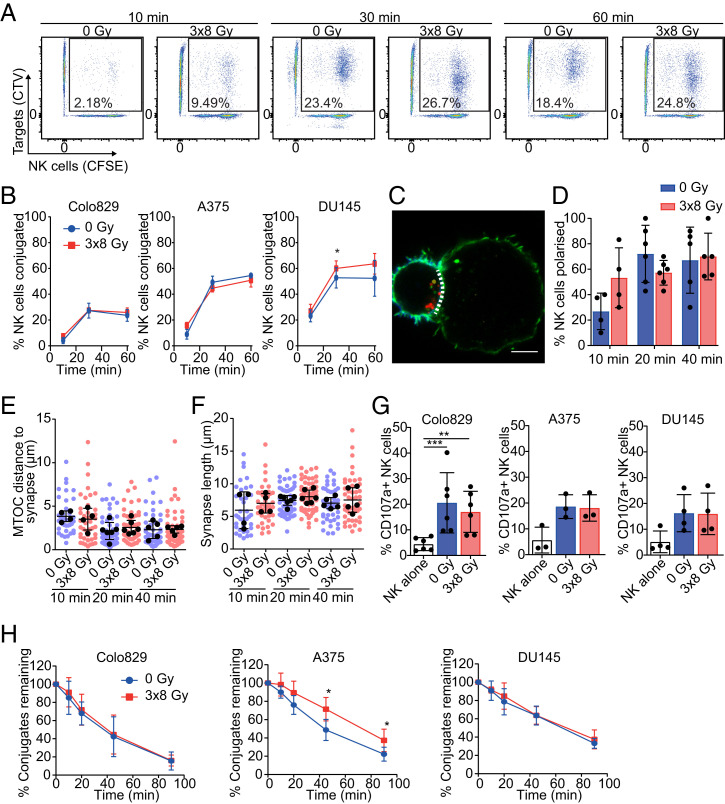
Cancer-cell irradiation does not affect the dynamics of the NK cell–cancer cell interaction. (*A* and *B*) NK-cell conjugation was quantified by incubation of dye-labeled NK cells with dye-labeled 0 Gy– or 3 × 8 Gy–treated cells for 10, 30, or 60 min. The percentage of double-positive conjugates was determined by flow cytometry. Representative flow cytometry plots of Colo829 cells (*A*) and quantification of Colo829, A375, and DU145 cells is shown (*B*) (*n* = 3). (*C*–*F*) Colo829 cells were incubated with NK cells for 10, 20, or 40 min and stained for CD56 (blue), pericentrin (yellow), actin (green), and perforin (red). Representative images show an NK cell (*Left*) and a target cell (*Right*) with a dotted line indicating the length of the immune synapse (*C*). (Scale bar, 5 µm.) The percentage of NK cells polarized per donor (distance of MTOC to immune synapse/diameter of cell <0.333) (*D*), the distance of the MTOC to immune synapse per conjugate (*E*), and the synapse length per conjugate (*F*) were calculated (*n* = 4 to 6). (*G*) NK-cell degranulation was quantified by measuring CD107a surface expression following a 5-h incubation with Colo829, DU145, and A375 cells (*n* = 3 to 6). (*H*) NK-cell detachment was quantified by incubation of NK cells with irradiated or nonirradiated Colo829, A375, or DU145 cells for 30 min prior to dilution in excess media (time = 0 min) to prevent further conjugation. The percentage of conjugates was determined at each time point, and conjugates remaining were quantified as a relative percentage to the conjugates at time 0 (*n* = 3). Means ± SD are indicated. Significance was determined using a paired *t* test (*B*, *D*–*F*, and *H*) or a repeated-measures one-way ANOVA (*G*). *P < 0.05, **P < 0.01, and ***P < 0.001.

To measure conjugation, the formation of double-positive events composed of carboxyfluorescein succinimidyl ester (CFSE)-labeled NK cells and CellTrace Violet (CTV)-labeled target cells was assessed by flow cytometry after 10-, 30-, or 60-min incubations. Conjugation was not affected by irradiation of Colo829, A375, or DU145 cells (Fig. 2 *A* and *B*). The ability of NK cells to polarize in response to irradiated cells was determined by imaging NK cell–cancer cell conjugates (Fig. 2 *C*–*F* and *SI Appendix*, Fig. 2). NK cells displayed strong polarization of the MTOC toward both irradiated cells and nonirradiated cells alike (Fig. 2 *C*–*E*). Furthermore, the length of the synapse, measured after 10, 20, and 40 min of coincubation, was not affected by irradiation of the target cell (Fig. 2*F*). NK-cell degranulation, determined by CD107a expression on the surface of NK cells following coincubation with Colo829, DU145, and A375 cells, was also unaffected (Fig. 2*G*). Lastly, we determined the ability of NK cells to detach from target cells, which is known to be important for the ability of NK cells to kill more than one target cell serially (11, 26). Although detachment from irradiated A375 cells was slightly reduced over a 90-min period, detachment of NK cells from Colo829 and DU145 cells was entirely unaffected by target cell irradiation (Fig. 2*H*). Thus, irradiation had no discernible effect on the overall dynamics of NK cell–cancer cell interactions.

**Fig. 3.**
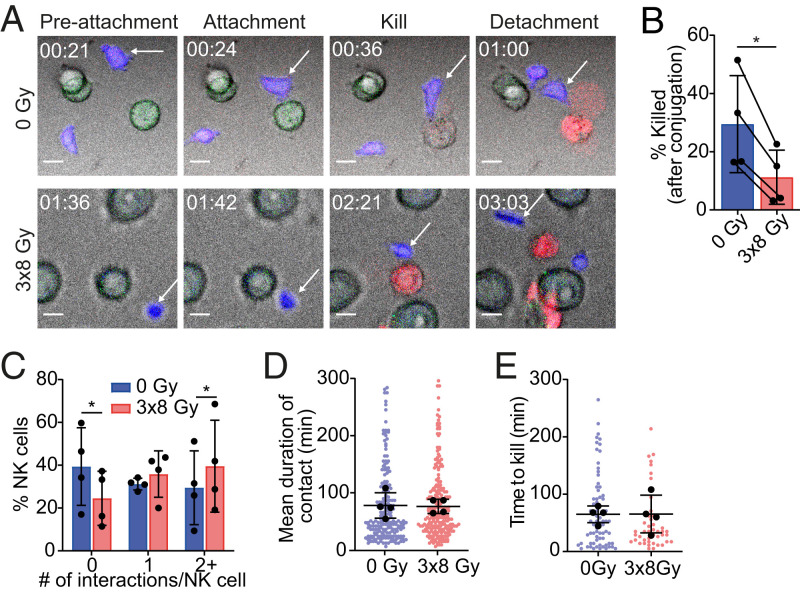
NK cells interact normally with irradiated cancer cells. Live-cell imaging was carried out over 5 h for NK cells incubated with untreated or 3 × 8 Gy–treated Colo829 cells in 350 × 350 µm microwells. (*A*) Representative images showing an NK cell (blue; white arrow) prior to attachment to a target cell (green), then subsequent attachment, killing (To-pro-3 entry; red), and detachment from the dead target cell. (Scale bar, 10 µm.) Time shown as hh:mm. (*B*–*E*) The percentage of conjugates resulting in a kill (*B*), the number of interactions per NK cell (*C*), the mean duration of each contact between an NK cell and target (*D*), and the time taken to kill after forming a conjugate (*E*) were recorded (*n* = 4). Black dots represent means of experiments, and colored dots represent individual events (*B*–*E*). Means ± SD are indicated. Significance was determined using a paired *t* test (*B*–*D*) or Mann–Whitney *U* test (*E*). *P < 0.05.

NK cell–cancer cell interactions were further studied at a single-cell level using live-cell imaging in 350 × 350 µm microwells over 5 h (Fig. 3*A*). Consistent with bulk assays (Fig. 1), irradiated cells died significantly less following interaction with an NK cell compared to nonirradiated cells (3 × 8 Gy: 11 ± 9%; 0 Gy: 30 ± 17%; Fig. 3*B*). Irradiation resulted in slightly more interactions with target cells by NK cells (mean number of interactions: 1.4 ± 0.6 [3 × 8 Gy] versus 1.1 ± 0.6 [0 Gy]) (Fig. 3*C*). However, the duration of each interaction, which was ∼75 min for both irradiated and nonirradiated cells, was unaffected by treatment (Fig. 3*D*). Likewise, the time taken to kill from conjugation was unaffected (Fig. 3*E*). Thus, changes to the dynamics of the NK cell–cancer cell interaction or reductions in NK-cell activation do not appear to explain the profound resistance of irradiated cancer cells to NK cell cytotoxicity.

### Irradiated Cancer Cells Are Resistant to Perforin.

**Fig. 4.**
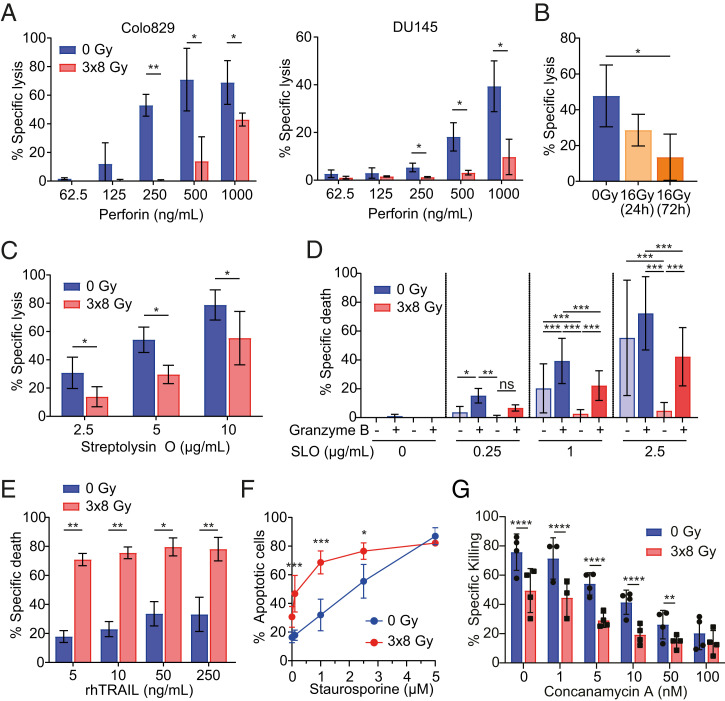
Radiotherapy induces resistance to perforin. (*A*) Colo829 and DU145 cells were treated for 15 min with native human perforin. Specific lysis was assessed using PI staining by flow cytometry (*n* = 3). (*B*) Colo829 cells were treated with 16 Gy for 24 or 72 h, prior to treatment with 500 ng/mL perforin for 15 min. Lysis was assessed as in *A* (*n* = 3). (*C*) Colo829 cells were treated with SLO for 15 min, and lysis was assessed as in *A* (*n* = 4). (*D*) Colo829 cells were treated with SLO and granzyme B for 4 h. Specific death was determined by annexin V/PI relative to baseline death (*n* = 4). (*E*) Colo829 cells were treated with recombinant human TRAIL (rhTRAIL) for 24 h, and specific death was determined as in *D* (*n* = 3). (*F*) Colo829 cells were treated with staurosporine for 24 h, and apoptosis was determined by annexin V/PI. (*G*) NK cells were treated for 2 h with concanamycin A, then incubated with 0 Gy or 3 × 8 Gy–treated Colo829 cells for 5 h. Specific killing was determined by annexin V/PI staining and normalized to control without NK cells (*n* = 4). Mean ± SD are indicated. Significance was determined using a paired *t* test (*A*, *C*, and *E*–*G*) or repeated-measures one-way ANOVA (*B* and *D*). *P < 0.05, **P < 0.01, ***P < 0.001, and ****P < 0.0001.

The preserved functionality and dynamics of NK-cell activation in response to irradiated cancer cells implies the presence of a cancer cell–intrinsic mechanism of resistance to cytotoxicity. NK cells employ either granule-mediated cytotoxicity, requiring perforin-mediated entry of granzyme B into target cells, or death receptor–mediated cytotoxicity (5). First, to determine whether or not irradiated cancer cells were susceptible to perforin, Colo829 and DU145 cells were treated with isolated human perforin protein. Lysis increased in a dose-dependent manner for nonirradiated cells (Fig. 4*A* and *SI Appendix*, Fig. 3*A*). However, radiotherapy led to a significant reduction in perforin-mediated lysis at 250 to 1,000 ng/mL for both Colo829 and DU145 cells. Approximately two- to fourfold the concentration of perforin was required to achieve a similar amount of death in irradiated cells as compared to nonirradiated cells. This difference in lysis was not due to differing kinetics, as increasing the perforin incubation time from 15 to 25 or 45 min did not increase lysis of either control or irradiated cells (*SI Appendix*, Fig. 3*B*). Resistance to NK-cell killing was previously observed to arise 72 h after irradiation with a single 16 Gy dose (Fig. 1*B*). In accordance with this, resistance to perforin-mediated lysis also arose primarily 72 h after irradiation (Fig. 4*B*). Likewise, treatment of Colo829 cells for 72 h with the cell-cycle inhibitors nocodazole and palbociclib also induced a profound resistance to perforin-induced lysis (*SI Appendix*, Fig. 1 *F* and *G*).

Perforin is a canonical member of the Membrane Attack Complex/Perforin (MACPF)/Cholesterol-Dependent Cytolysin (CDC) superfamily (27). Proteins within the MACPF/CDC superfamily, which also contains bacterial pore-forming toxins such as streptolysin O (SLO), all share a distinctive structure and mechanism of pore formation (27). Therefore, to test whether or not irradiation-induced resistance to pore-forming proteins more generally, we treated Colo829 cells with purified SLO and found that irradiated cells were also significantly more resistant to SLO-induced lysis (Fig. 4*C*).

To test whether sensitivity to granzyme B is affected by irradiation, we treated Colo829 cells with granzyme B and varying concentrations of SLO to facilitate granzyme B entry in an analogous manner to perforin. In the absence of SLO, granzyme B did not induce cell death in either irradiated or nonirradiated cells (Fig. 4*D*). When SLO was present at sufficiently high concentrations, granzyme B significantly increased death in both irradiated and nonirradiated cells, suggesting that irradiated cells remain highly susceptible to granzyme B. However, a greater amount of SLO was required to induce granzyme B–mediated death in irradiated cells (1 µg/mL) compared to nonirradiated (0.25 µg/mL). Overall, at all concentrations tested, SLO and granzyme B induced a significantly lower degree of death in irradiated cells compared to nonirradiated cells.

NK-cell cytotoxicity can also be facilitated by death-receptor signaling, which is known to increase following radiotherapy (28–31). Here, we found that Colo829, DU145, and A375 cells treated with 3 × 8 Gy were significantly more susceptible to apoptosis following treatment with recombinant TRAIL protein for 24 h (Fig. 4*E* and *SI Appendix*, Fig. 3*C*). Furthermore, irradiated Colo829 cells were susceptible to death induced by a cytotoxic drug, staurosporine (Fig. 4*F*). Overall, this demonstrates that radiotherapy is not generally selecting for a subset of cells resistant to apoptosis but that irradiation specifically increases resistance to pore-forming proteins.

To directly test if perforin resistance accounts for resistance to NK-cell cytotoxicity following radiotherapy, NK cells were depleted of perforin using concanamycin A (*SI Appendix*, Fig. 3*D*) (32). Concanamycin A induced a concentration-dependent decrease in cytotoxicity against both irradiated and nonirradiated Colo829 cells (Fig. 4*G*). At a particularly high concentration of concanamycin A, the difference in cytotoxicity against irradiated and nonirradiated cancer cells was lost. Thus, granule-mediated cytotoxicity accounts for the majority of NK-cell killing in these experiments and the reduced sensitivity of irradiated cancer cells to NK-cell cytotoxicity is dependent upon perforin.

### Perforin Binds but Fails to Form Functional Pores in Membranes of Irradiated Cells.

**Fig. 5.**
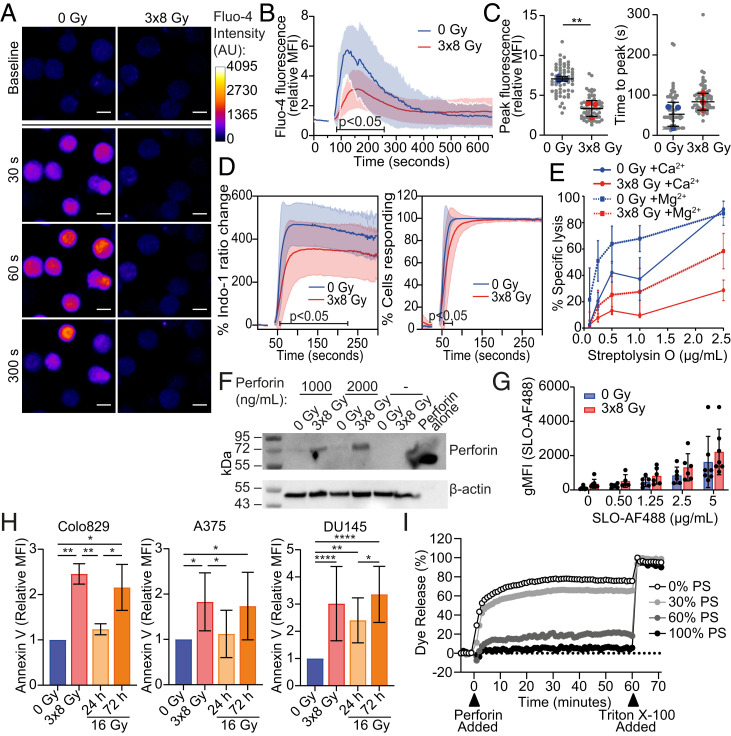
Functional pore formation is inhibited on irradiated cell membranes. (*A*–*C*) Colo829 cells were stained with fluo-4 and imaged by confocal microscopy to determine calcium flux after addition of 20 µg/mL recombinant perforin at 60 s. Representative images of fluo-4 fluorescence shown at baseline and varying times after perforin addition (*A*). (Scale bar, 10 µm.) The change in fluo-4 fluorescence relative to baseline over time (*B*), peak fluo-4 fluorescence relative to baseline, and the time taken to reach peak were calculated for 20 cells per condition (*n* = 3) (*C*). Solid line and shaded area indicate mean and SD, respectively. Gray and colored dots indicate individual cells and means of experiments, respectively. (*D*) Colo829 cells were stained with indo-1, and calcium flux was quantified using flow cytometry after addition of 0.5 µg/mL SLO at 30 s. The change in indo-1 fluorescence ratio relative to baseline and the percentage of cells responding with increased calcium concentration above baseline were determined. (*E*) Colo829 cells were treated with SLO in the presence of 2 mM CaCl_2_ or MgCl_2_ in PBS, and lysis was assessed using PI staining by flow cytometry (*n* = 3). (*F*) Colo829 cells were treated with 1,000 or 2,000 ng/mL native perforin for 15 min, and binding of perforin was assessed by Western blot. β-actin was used as a loading control. (*G*) Colo829 cells were treated with AF488-conjugated SLO for 15 min, and binding was assessed by flow cytometry. (*H*) Cancer cells treated with radiotherapy (0 Gy, 3 × 8 Gy, or 24 to 72 h after 16 Gy) were stained with annexin V to quantify surface phosphatidylserine and analyzed by flow cytometry. MFI is shown relative to 0 Gy. (*I*) Liposomes composed of phosphatidylcholine and 0, 30, 60, or 100% phosphatidylserine (PS) and containing 5,6-carboxyfluorescein dye were treated with 20 µg/mL recombinant human perforin. Lysis of liposomes was determined by dye release tracked over time (*n* = 3). Triton X-100 was added at 60 min to induce total lysis. Mean ± SD are indicated. Significance was determined using a two-way ANOVA (*B* and *D*), unpaired *t* test (*C*), paired *t* test (*G*), or one-way ANOVA (*H*). *P < 0.05, **P < 0.01, and ****P < 0.0001.

Presence of a functional perforin or SLO pore in the membrane is marked by the flow of ions, such as calcium, across the membrane (33, 34). Therefore, to more directly investigate the ability of pore-forming proteins to functionally affect target cells, we tracked the intracellular calcium flux following exposure to perforin and SLO. In nonirradiated Colo829 cells, a sublytic dose of perforin triggered a rapid increase in intracellular calcium concentration (Fig. 5 *A* and *B*). At the same concentration, perforin also induced a calcium flux in 3 × 8 Gy–treated Colo829 cells, but the magnitude of the peak was significantly lower (Fig. 5 *A*–*C*). The time to peak calcium concentration was not significantly different (Fig. 5*C*). Upon exposure to a higher lytic dose, a greater calcium flux was also observed in nonirradiated cells (*SI Appendix*, Fig. 4*A*). Similar to perforin, SLO induced a rapid increase in calcium concentration in both irradiated and nonirradiated Colo829 cells (Fig. 5*D* and *SI Appendix*, Fig. 4*B*). Again, the magnitude of calcium flux was significantly lower in irradiated cells compared to nonirradiated cells. This reduction in both perforin- and SLO-induced calcium flux is consistent with a deficiency in pore formation.

In contrast, irradiated cells were more responsive to ionomycin, indicating that intracellular calcium flux was not limited in any general way, and there was no enhancement in the recovery of calcium homeostasis (*SI Appendix*, Fig. 4*C*). Thus, resistance to pore-forming proteins in irradiated cells is reflected in both reduced lysis and a reduced calcium flux.

Pore-induced calcium flux is critical for the induction of a repair process that removes membrane pores and prevents lytic death (33–35). Such membrane repair can be inhibited by depleting extracellular calcium. Perforin itself requires calcium to function, but SLO does not (27). Therefore, we used SLO to test the contribution of calcium-induced membrane repair to the resistance of irradiated cells to pore-forming proteins. When Colo829 cells were treated with SLO in the absence of extracellular calcium, lysis of both irradiated and nonirradiated cells was increased (Fig. 5*E*). However, irradiated cells remained more resistant to SLO compared to nonirradiated cells, suggesting that calcium-mediated membrane repair cannot underlie the resistance of irradiated cells to pore-forming proteins. If that were the case, then the effect of irradiation would be lost in the absence of calcium.

The formation of functional perforin and SLO pores requires the binding of monomers to the membrane prior to oligomerization and pore formation (36). Therefore, we tested the binding of perforin and SLO to membranes of irradiated cells. We observed binding of perforin to both irradiated and nonirradiated Colo829 cells (Fig. 5*F*). Critically, irradiated cells did not bind less perforin compared to nonirradiated cells. Likewise, when Colo829 cells were incubated with fluorescently tagged SLO, binding increased in a concentration-dependent manner, but there was no difference in its binding to irradiated compared to nonirradiated cells (Fig. 5*G* and *SI Appendix*, Fig. 4*D*). Importantly, we observed that equivalent levels of fluorescent SLO binding induced far greater lysis in nonirradiated cells compared to irradiated cells (*SI Appendix*, Fig. 4*E*). SLO binding is dependent on membrane cholesterol (27). Therefore, we confirmed the specificity of fluorescent SLO binding by depleting cholesterol from the membrane using methyl-β-cyclodextrin, which entirely abolished SLO binding (*SI Appendix*, Fig. 4*F*). These data indicate that the binding of perforin and SLO monomers to irradiated cell membranes is not affected. Instead, assembly of a functional pore is impaired by irradiation.

A perforin-induced calcium flux was also significantly reduced in cancer cells treated with nocodazole and palbociclib to inhibit the cell cycle (*SI Appendix*, Fig. 5 *A*–*C*). As with irradiation, binding of fluorescent SLO to nocodazole- or palbociclib-treated cells was similar despite reduced susceptibility to SLO-induced lysis (*SI Appendix*, Fig. 5 *D* and *E*). Thus, similar to irradiation, pharmaceutically driven cell-cycle arrest also imposes resistance to pore formation.

To more directly test for impairment in functional pore formation on irradiated cell membranes, we tracked the entry of a small fluorescent dye, To-pro-3, and a large 150-kDa fluorescent dextran into cells over time upon treatment with perforin. Small dyes, such as To-pro-3, can enter directly through perforin pores in membranes, whereas large dextrans cannot (37, 38). Therefore, entry of To-pro-3 alone marks pore formation and entry of both To-pro-3 and dextran marks loss of membrane integrity and cell lysis.

**Fig. 6.**
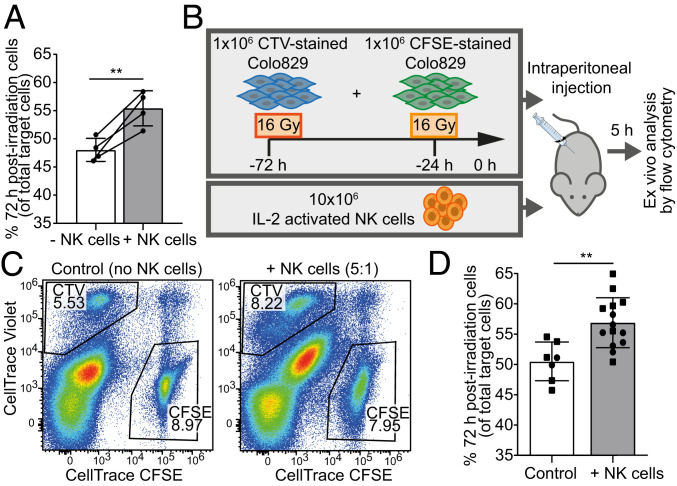
Radiotherapy causes resistance to NK-cell cytotoxicity in vivo. (*A*) Colo829 cells were treated with 16 Gy either 24 or 72 h prior, stained with CFSE and CTV, respectively, and mixed at a 1:1 ratio. Targets were incubated with human NK cells at a 5:1 E:T ratio for 5 h. The proportion of 72-h-postirradiation cells of total target cells is shown (*n* = 4). (*B*–*D*) A total of 1 × 10^6^ 72-h-postirradiation Colo829 cells and 1 × 10^6^ 24-h-postirradiation Colo829 cells were stained with CTV and CFSE, respectively, and injected into the peritoneum of NSG mice with 10 × 10^6^ IL-2–activated human NK cells. After 5 h, peritoneal effluent was analyzed by flow cytometry to determine the percentage of 72 h postirradiation cells of total target cells. Schematic of experiment (*B*) and representative FACS plot (*C*) are shown. The experiment was carried out in two independent cohorts (squares and circles; control: *n* = 7, +NK cells: *n* = 14) (*D*). Mean ± SD are indicated. Significance was determined using a paired *t* test (*A*) and unpaired *t* test (*D*). **P < 0.01.

Treating Colo829 cells with a lytic concentration of recombinant human perforin (40 µg/mL) resulted in rapid influx of To-pro-3 followed by entry of the 150 kDa dextran after a ∼60-s delay, indicating rapid pore formation followed by lysis (*SI Appendix*, Fig. 6*A*). In contrast, slower and reduced influx of To-pro-3 was triggered in irradiated cells with no entry of 150 kDa dextran observed, consistent with reduced pore formation and no lysis. Treatment with a sublytic perforin dose (20 µg/mL) induced some influx of To-pro-3 into both nonirradiated and irradiated cells, but influx into irradiated cells was far less (*SI Appendix*, Fig. 6*B*). With this sublytic dose of perforin, 150 kDa dextran did not enter irradiated or nonirradiated cells. Overall, this data further establishes that perforin pore formation is reduced on irradiated cancer cells compared to nonirradiated cells.

Cytotoxic lymphocytes have been previously shown to protect themselves against perforin-induced self-harm through the externalization of negatively charged membrane phosphatidylserine, which prevents the assembly of perforin monomers into pores (39). Therefore, we investigated whether phosphatidylserine externalization on irradiated cancer cells contributes to protection against perforin. Treatment of Colo829, A375, and DU145 with 3 × 8 Gy radiotherapy resulted in a significant increase in surface phosphatidylserine (Fig. 5*H* and *SI Appendix*, Fig. 4*G*). Treatment of cancer cells with a single dose of 16 Gy 72 h prior, but not 24 h, also resulted in phosphatidylserine externalization (Fig. 5*H*). Likewise, treatment of Colo829 cells with cell-cycle inhibitors nocodazole and palbociclib led to a significant increase in surface phosphatidylserine after 72 h but not after 24 h (*SI Appendix*, Fig. 4*H*). Therefore, phosphatidylserine externalization coincides with perforin resistance.

To test if phosphatidylserine can indeed inhibit the lytic ability of perforin, we produced liposomes composed of phosphatidylcholine and varying molar concentrations (0, 30, 60, and 100%) of phosphatidylserine and containing 5,6-carboxyfluorescein. The 5,6-carboxyfluorescein release can be recorded as a measure of liposome lysis (40). Perforin lytic ability was found to be significantly reduced against liposomes composed of 60% and 100% phosphatidylserine (Fig. 5*I* and *SI Appendix*, Fig. 4*I*). Therefore, phosphatidylserine can prevent pore formation by perforin, and its externalization on irradiated cancer cells is concurrent with perforin resistance.

### Radiotherapy Induces Resistance to NK-Cell Cytotoxicity In Vivo.

Treatment with radiotherapy in vivo can lead to bystander effects, whereby signals released from irradiated cells influence neighboring cells (41). Therefore, we next tested whether cells, which are susceptible to NK-cell cytotoxicity when cultured alone, remain susceptible when cultured with cells exhibiting radiation-induced resistance. To do this, we mixed together a 1:1 ratio of Colo829 cells treated with 16 Gy either 24 h or 72 h prior and then incubated these cells with NK cells for 5 h. In the presence of NK cells, we observed a significant increase in the number of 72 h postirradiation cells relative to 24 h postirradiation cells (48 ± 2% to 55 ± 3% of total targets) due to enhanced depletion of the susceptible 24-h-postirradiation cells (Fig. 6*A* and *SI Appendix*, Fig. 5*A*). This further establishes that cancer cells are resistant to NK-cell cytotoxicity 72 h, but not 24 h, after treatment with radiotherapy. Additionally, this suggests that bystander effects between susceptible and resistant cells do not contribute to resistance following radiotherapy.

Next, to test whether radiotherapy also leads to resistance against NK-cell cytotoxicity in vivo, we differentially dye labeled 24-h- and 72-h-postirradiation Colo829 cells and injected a 1:1 ratio of these cells intraperitoneally into NSG (NOD scid gamma) mice lacking mature T cells, B cells, and NK cells (Fig. 6*B*). We simultaneously injected peripheral blood human NK cells, and 5 h later, the peritoneal cavity was flushed and the effluent analyzed by flow cytometry. We observed a significant increase in the percentage of 72-h-postirradiation cells of total targets from 50 ± 3 to 56 ± 4% (Fig. 6 *C* and *D* and *SI Appendix*, Fig. 5*B*). Because cells at this stage posttreatment are not proliferating, this must be due to enhanced NK cell–mediated cytotoxicity against 24-h-postirradiation cells relative to 72-h-postirradiation cells. Thus, radiation induces resistance to NK cell–mediated killing in vivo as well as in vitro.

### Radiotherapy Induces Resistance to Cytotoxic T-Cell Killing.

**Fig. 7.**
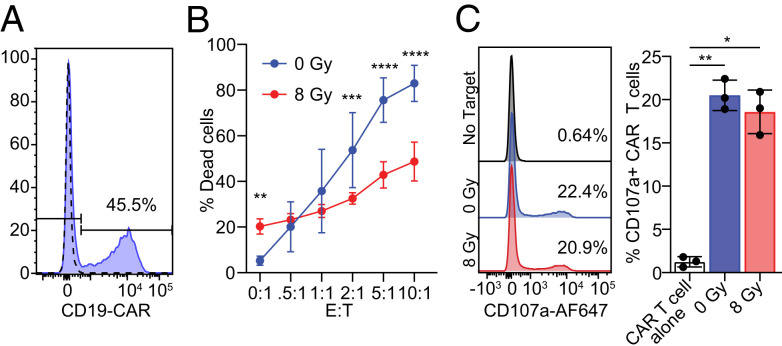
Radiotherapy induces resistance to CAR T-cell cytotoxicity. (*A*) CD19 CAR expression on transduced T cells. Dotted line indicates mock transduced cells. (*B*) CD19-specific CAR T cells were incubated for 5 h at varying effector-to-target ratios (E:T) with Daudi cells treated with 8 Gy 72 h prior. Lysis of Daudi cells was determined by annexin V/PI staining by flow cytometry (*n* = 4). (*C*) CD19-specific CAR T-cell degranulation was quantified by measuring CD107a surface expression following a 5-h incubation with Daudi cells treated with 0 or 8 Gy 72 h prior. Representative flow cytometry plots with percentage of CD107a+ CAR T cells shown on left. Quantification of CD107a expression on CAR T cells shown on right (*n* = 3). Mean ± SD are indicated. Significance was determined using a two-way ANOVA (*A*) and one-way ANOVA (*B*). *P < 0.05, **P < 0.01, ***P < 0.001, and ****P < 0.0001.

In addition to NK cells, granule-mediated cytotoxicity is also utilized by cytotoxic T cells. Therefore, we next investigated the susceptibility of irradiated cancer cells to T-cell killing using CD8+ T cells expressing a CD19-specific chimeric antigen receptor (CAR) (Fig. 7*A*). CD19-expressing Daudi cells were treated with a single dose of 8 Gy 72 h prior to incubation for 5 h with CD19 CAR T cells at varying effector-to-target ratios. Irradiation of Daudi cells resulted in a significant decrease in T-cell killing, similar to observations with NK cells (Fig. 7*B*). Additionally, as was observed with NK cells, this reduction in cytotoxicity was not a result of reduced T-cell activation, since T cells degranulated to a similar extent in response to irradiated and nonirradiated target cells (Fig. 7*C*). Thus, irradiation-induced resistance to perforin results in a significant reduction in killing by cytotoxic lymphocytes of both the innate and adaptive immune systems.

## Discussion

The effects of radiotherapy on antitumor immune responses are increasingly understood, but little is known about the impact of irradiation on the interaction between NK cells and cancer cells specifically. Unexpectedly, we found that treatment of multiple types of cancer cells with clinically relevant doses of radiation resulted in significant resistance to NK-cell cytotoxicity in a time-dependent manner. Examination of the dynamics of NK cell–cancer cell interactions did not reveal any significant effects of target cell irradiation. Instead, reduced susceptibility to lysis was caused by a profound resistance to perforin. This resistance to perforin was also reflected in reduced pore-induced calcium flux, while binding of monomers appeared unaffected. This indicates that perforin fails to form functional multimeric membrane pores in irradiated cancer cells. Resistance to perforin coincided with an increase in surface phosphatidylserine on irradiated cancer cells, and in model synthetic liposomes this lipid can inhibit perforin pore formation. In addition, radiotherapy-treated cancer cells were also resistant to cytotoxicity by CAR T cells.

Radiotherapy, a major inducer of cellular stress, has been reported to drive an increase in the expression of stress-inducible NK cell–activating ligands, such as ULBP1-3, MICA/B, and B7-H3, on the surface of cancer cells primarily through activation of the ataxia-telangiectasia mutated (ATM)/ATM- and Rad3-Related (ATR) DNA damage response pathway (14–18). Conversely, radiotherapy can also increase the expression of class I major histocompatibility complex (MHC) molecules, which act as inhibitory ligands for NK cells (31, 42). Nevertheless, while there may be complex effects on ligand expression as a result of radiotherapy, we did not observe any effect on NK-cell activation or dynamics here. Instead, we found that a potent consequence of radiotherapy for NK cell–mediated attack is a resistance to perforin. This resistance to perforin also resulted in reduced killing by T cells. We did observe an increase in susceptibility to death receptor–mediated cytotoxicity, but depletion of perforin from NK cells revealed that killing was primarily performed by granule-dependent cytotoxicity. Previous work suggests that NK cells predominantly carry out serial killing of target cells via granule-mediated cytotoxicity and kill only later via the death-receptor pathway (26). Therefore, perforin resistance has the capacity to severely constrain NK-cell and T-cell cytotoxicity following radiotherapy.

Perforin is a crucial component of granule-mediated cytotoxicity and may lead to cell death in two ways. At higher concentrations, necrosis can be caused by osmotic lysis through membrane pores. At lower concentrations, regulated cell death—generally apoptosis—is caused by perforin-mediated translocation of granzymes from the immune synapse to the target cell cytoplasm (34, 35, 37). Both necrosis and apoptosis can contribute to overall death induced by NK cells, though the relative contribution of each to NK-cell killing is not clear (26, 43). The transport of granzymes across the membrane is not fully understood but is thought to require either endocytosis triggered by perforin-induced calcium flux (34, 35) or direct delivery through the perforin pore (37). We found that cancer-cell necrosis via direct lysis by perforin—or a structurally and mechanistically similar bacterial pore-forming toxin, SLO—was significantly reduced following irradiation. Irradiated cells remained susceptible to granzyme B, but a significantly higher concentration of pore-forming SLO was required for granzyme B to be taken up sufficiently to induce death. We also observed reduced transit of fluorescent dye directly through perforin pores in irradiated cell membranes. Overall, these results indicate that the resistance to lymphocyte cytotoxicity following cancer-cell irradiation can result from both a loss in direct lysis by perforin and reduced uptake of granzyme B.

Resistance to perforin likely plays an important role in cancer etiology, since significant variation between patients in the susceptibility of leukemic cells to perforin has been observed (44, 45). Several mechanisms have been described that can mediate protection against perforin, including secretion of a lysosomal cysteine protease, cathepsin B (46), modulation of membrane tension (47), and changes to membrane composition (39, 48). These inhibitory mechanisms can affect perforin at any stage of its action, including degradation of perforin upon secretion, insertion of monomers into the membrane, oligomerization into a functional pore, and membrane repair. We found that neither perforin nor SLO binding was reduced on irradiated cancer cells and that membrane repair was not responsible for differences between the susceptibility of irradiated and nonirradiated cancer cells. Instead, our data are consistent with a failure of membrane-bound monomers to coalesce into a functional multimeric pore on irradiated cells. Phosphatidylserine externalization has been proposed as a protective mechanism against self-harm by perforin in cytotoxic lymphocytes (39). It was also shown that phosphatidylserine on planar lipid bilayers does not prevent perforin binding but induces the formation of perforin plaques rather than complete pores (39). We found that surface phosphatidylserine is significantly increased 72 h after irradiation or induction of cell-cycle arrest, which coincides with the timing of perforin resistance. This may represent an enrichment of a small population of cells by radiotherapy or may be caused by radiation directly. We also showed that the lytic activity of perforin is profoundly impaired on liposomes composed of high levels of phosphatidylserine. This provides a potential mechanism by which cancer cells become resistant to perforin pore formation following radiotherapy or prolonged cell-cycle arrest. The cause of enhanced phosphatidylserine externalization requires further study to identify possible druggable targets.

Immunotherapies are being increasingly combined with radiotherapy, and an optimal protocol is important to establish. Hypofractionated radiotherapy schedules, which consist of a smaller number of high doses, are often regarded as being particularly immunogenic and effective in combination with immunotherapy (19, 20). However, our data suggest that resistance to lymphocyte cytotoxicity, including killing via ADCC, could significantly limit the success of a broad range of radiotherapy–immunotherapy combinations that target either adaptive or innate immunity. The timing is crucial here. We discovered that a critical 72-h period exists following irradiation in which cancer cells remain susceptible to cytotoxicity. Following this period, cells become highly resistant for approximately 2 wk. Therefore, careful consideration needs to be given to sequencing of radiotherapy and immunotherapy combinations.

In addition to radiotherapy, we showed that resistance to perforin can also be triggered using pharmaceutical compounds that inhibit the cell cycle. Drugs that target the cell cycle are currently in clinical trials, and some, such as palbociclib, have already been approved for the treatment of breast cancer (49). Like radiotherapy, these drugs are capable of eliciting an antitumor immune response (50), but the timing of this and whether or not there is a transient blind spot to perforin-mediated killing is not known.

Much of the research on antitumor immunity and immunotherapies has focused on the response of immune cells to cancer cells. Here, we show that cancer-cell susceptibility to cytotoxicity is a crucial aspect to take into consideration, as this can be significantly altered by a primary form of cancer treatment, radiotherapy. Moreover, understanding radiotherapy-induced resistance to lymphocyte cytotoxicity could drive the development of novel treatment combinations and lead to a better outcome for patients.

## Materials and Methods

### Cell Culture.

All cells were cultured at 37 °C and 5% CO_2_. Colo829 human melanoma cells (51), DU145 human prostate cancer cells (52), and Daudi B-cell lymphoma cells (53) transfected to express beta-2-microglobulin (Daudi-β2M) were maintained in Roswell Park Memorial Institute (RPMI)-1640 (Sigma) with 2 mM L-glutamine (Gibco). A375 (54) and WM-266.4 human melanoma cells (55) were maintained in high-glucose Dulbecco’s Modified Eagle’s Medium (DMEM; Sigma). A549 human lung cancer cells (54) and PC-3 prostate cancer cells (56) were maintained in Ham’s F-12 (Sigma) with 2 mM L-glutamine. All media were supplemented with 10% fetal calf serum (FCS; Sigma) and 50 U/mL penicillin–streptomycin (Sigma). All cell lines were routinely tested and found to be negative for mycoplasma using a PCR-based kit (PromoCell).

### Irradiation and Drug Treatments.

For in vitro assays, cells were treated with X-ray irradiation (0.795 Gy/min) using an MXR-320/36 X-ray tube assembly (320 kV; Comet AG). For in vivo assays, cells were irradiated in vitro using an XSTRAHL CIX3 irradiator (300 kV) prior to inoculation. For fractionated radiotherapy (3 × 8 Gy and 5 × 2 Gy), 8-Gy or 2-Gy doses were given 24 h apart for 3 or 5 d, respectively. Nonirradiated (0 Gy) cells received simultaneous mock irradiation. For drug treatments, cells were treated with 0.5 µM nocodazole (Sigma) or 5 µM palbociclib (Sigma) with fresh media and drug added at 24 h intervals. Unless otherwise stated, cells were incubated for 24 h after drug treatment or irradiation and then detached using trypsin-ethylenediaminetetraacetic acid (EDTA; Sigma) prior to carrying out assays. For concanamycin A treatment of NK cells, concanamycin A (Tocris) was added in complete RPMI-1640 for 2 h at 37 °C and then washed out before use of NK cells.

### Isolation of Primary Human NK Cells.

Peripheral blood leukocyte cones from healthy human donors were acquired from the National Health Service blood service under ethics license REC 05/Q0401/108 (University of Manchester). Peripheral blood mononuclear cells (PBMCs) were isolated using density gradient centrifugation with Ficoll Paque (GE Healthcare). NK cells were isolated by negative selection (NK Cell Isolation Kit; Miltenyi Biotec) and cultured in DMEM with 30% Ham’s F-12, 10% human serum (Sigma), 1% nonessential amino acids (Sigma), 1 mM sodium pyruvate (Sigma), 2 mM L-glutamine, 50 U/mL penicillin–streptomycin, and 50 μM 2-mercaptoethanol (Gibco). Media were supplemented with 200 U/mL interleukin-2 (Roche) on day 0, and NK cells were used in assays on day 6.

### Production of CAR T Cells.

CD19-specific CAR T cells were generated using a pMP71 retroviral vector kindly provided by David Gilham (The University of Manchester, United Kingdom) (57). PBMCs were isolated from leukocyte cones as described in *Isolation of Primary Human NK Cells*. PBMCs were cultured for 48 h in T-cell medium (RPMI with 10% FCS, 2 mM L-glutamine, 50 U/mL penicillin–streptomycin, and 25 mM HEPES [Sigma]) containing 50 ng/mL anti-CD3 (Biolegend, clone: OKT3) and anti-CD28 (R&D Systems, clone: 37407) antibodies and 200 U/mL IL-2 resulting in a 99% CD3+ cell population. For transduction, 6-well plates (CytoOne, STARLAB) were coated with 20 µg/mL RetroNectin (Takara Bio) and incubated overnight at 4 °C. Wells were blocked with 2% BSA (bovine serum albumin; Sigma) in phosphate buffered saline (PBS) for 30 min at room temperature (RT) and washed twice with PBS. Anti-CD19 CAR retrovirus from PG13 cells was added, and plates were centrifuged at 37 °C for 2 h and then washed twice with PBS. A total of 2 × 10^6^ T cells were added per well with 200 U/mL IL-2. Plates were incubated overnight at 37 °C. The transduction process was repeated the following day, with T cells undergoing two successive rounds of transduction. Transduced T cells were then harvested for use in assays.

### Killing Assays.

Lymphocytes were labeled using the CTV Cell Proliferation Kit (Invitrogen). Lymphocytes were then cultured in complete RPMI-1640 (2 mM L-glutamine, 10% FCS, and 1% penicillin–streptomycin) with 5 × 10^4^ cancer cells for 5 h at 37 °C. To quantify cell death, cells were detached using trypsin-EDTA, stained with annexin V-APC (Biolegend) in annexin V binding buffer (2.5 mM CaCl_2_, 140 mM NaCl, and 10 mM HEPES) for 15 min at RT, and washed once with binding buffer. A total of 1 µg/mL propidium iodide (PI) was added prior to immediate analysis by flow cytometry (BD FACS Canto II or LSR-II or Fortessa x20). Data analysis was performed using FlowJo V10 (BD Biosciences).

To measure ADCC, Daudi-β2M cells were incubated with 10 μg/mL anti-CD20 antibody (rituximab; a kind gift from GlaxoSmithKline) or an isotype-matched control antibody for 30 min at 37 °C prior to addition of NK cells at a 1:1 ratio for 2 h, after which cell death was quantified using annexin V/PI as previously.

For analysis of long-term susceptibility to NK-cell cytotoxicity following irradiation, cells were treated with three doses of 8 Gy. A total of 24 h after irradiation, 2.5 × 10^5^ cells per well were plated in 6-well plates. Cells were counted and replated at 2- to 3-d intervals, and counts are reported as the percentage of cells seeded relative to the original number of cells seeded in each well divided by days since seeding. Killing assays were performed at times denoted, as described above.

For coculture experiments, Colo829 cells were treated with 16 Gy either 24 or 72 h prior to the experiment. A total of 24 and 72 h postirradiation, cells were labeled with CellTrace CFSE and CTV, respectively. A 1:1 ratio of 24- and 72-h-postirradiation cells (2.5 × 10^4^ cells each) was incubated with NK cells (2.5 × 10^5^) for 5 h at 37 °C. The percentage of 72-h-postirradiation cells relative to total target cells was determined by flow cytometry.

### Cell-Cycle Analysis.

Cells were fixed with 70% ethanol (prechilled to −20 °C) for 30 min at 4 °C. Cells were then washed twice in PBS and treated with RNase (PureLink RNase A, Invitrogen; 20 μg/mL) and stained with 20 μg/mL PI. Cells were analyzed by flow cytometry.

### Phosphorylated H2AX Detection.

A total of 1 × 10^5^ treated Colo829 cells were incubated in wells of a 0.01% poly-L-lysine (PLL; Sigma)-coated eight-chambered glass coverslip (#1.5 Lab-Tek II; Nunc) for 1 h at 37 °C. Cells were washed with PBS, fixed with 4% paraformaldehyde (PFA) for 20 min, permeabilized with 0.1% Triton X-100 for 10 min, washed with PBS, and blocked with blocking buffer (PBS + 3% BSA) for 1 h, all at RT. Staining was performed using anti–γH2AX-AF647 antibody (1 µg/mL; clone: 2207D; R&D Systems), AF488-conjugated wheat germ agglutinin (5 µg/mL; Invitrogen), and Hoechst 33342 (NucBlue Live Ready Probes; Molecular Probes) for 1 h at RT. Cells were washed with PBS and imaged by confocal microscopy (Leica TCS SP8; Leica Microsystems) using a 100×/1.40 numerical aperture (NA) oil immersion objective with excitation using 405-nm, 488-nm, and 647-nm laser lines. Images were analyzed using ImageJ, and DNA damage was quantified by counting the number of γ-H2AX foci within the nucleus of each cell.

### Degranulation Assay.

Cancer cells were labeled with the CTV Cell Proliferation Kit and cultured with lymphocytes at a 1:1 ratio for 5 h at 37 °C in complete RPMI-1640 containing GolgiStop (1/1000; BD Biosciences) and AF647-labeled anti–LAMP-1 antibody (4 µg/mL; H4A3; Biolegend) or isotype-matched control (IgG1κ-AF647; MOPC-21; Biolegend). Cells were washed with PBS, blocked in 2% human serum (Sigma) for 30 min at 4 °C, and stained with a dead cell marker (Zombie NIR Fixable Viability Kit; Biolegend) and AF647-labeled anti–LAMP-1 antibody (4 µg/mL) or isotype-matched controls for 40 min at 4 °C. Cells were then fixed with 4% PFA for 30 min at 4 °C and analyzed by flow cytometry.

### Conjugation and Detachment Assay.

NK cells and cancer cells were stained with different dyes (CTV/CFSE Cell Proliferation kits; Invitrogen). A total of 1 × 10^5^ NK cells were mixed with 2 × 10^5^ target cells in a volume of 200 μL complete RPMI-1640. For conjugation assays, cells were incubated at 37 °C for 10, 30, or 60 min and fixed in 2% PFA for 30 min at 4 °C. For detachment assays, cells were incubated for 30 min and then diluted with 10 mL complete RPMI-1640 and rotated gently for 0, 10, 20, 45, and 90 min at 37 °C followed by fixation in 2% PFA at 4 °C for 30 min. Cells were analyzed by flow cytometry (FlowJo V10), and the percentage of NK cells conjugated was determined by quantifying the number of double-positive events as a proportion of the total number of NK cells.

### MTOC Imaging.

NK cell–cancer cell conjugates were formed by incubating NK cells with cancer cells for 10, 20, or 40 min before fixation with 4% PFA/PBS for 20 min at RT. Conjugates were permeabilized with 0.1% Triton X-100 (Sigma) for 10 min at RT, blocked with 3% BSA in PBS for 1 h at RT, and stained with anti-pericentrin antibody (1 µg/mL; ab4448; Abcam) in blocking buffer for 2 h at 4 °C. Conjugates were then washed with PBS, stained for 1 h at RT with AF568-conjugated goat anti-rabbit secondary antibody (5 µg/mL; Invitrogen), AF647-conjugated anti-perforin antibody (0.625 μg/mL; dG9; Biolegend), AF488-labeled phalloidin (10 µg/mL; Invitrogen), and BV421-conjugated anti-CD56 antibody (1.5 μg/mL; HCD56; Biolegend), washed with PBS, and transferred to a PLL-coated eight-chambered glass coverslip. Imaging was performed by confocal microscopy (SP8, Leica Biosystems) using a 63×/1.40 NA oil immersion objective with excitation using 405-, 488-, 568-, and 647-nm laser lines. Images were analyzed using ImageJ (58). The distance of the immune synapse to the MTOC and back of the cell was measured manually. The length of the immune synapse was quantified by measuring the area of contact between an NK cell and target cell based on actin staining at the z-plane in which contact was greatest.

### Live-Cell Imaging.

Live imaging of NK cell–cancer cell interactions was performed using a silicon glass chip with 350 × 350 μm microwells (59). Sterile chips were coated with 100 μg/mL fibronectin (Sigma) for 45 min at 37 °C and washed with complete media. NK cells and target cells were stained with 0.64 μM calcein red-orange and 0.3 μM calcein green (Thermo Fisher), respectively, for 20 min at 37 °C. Target cells and NK cells were added to microwells in complete media containing 1 μM To-pro-3 (Thermo Fisher). Microwells were imaged at 3-min intervals for 5 h at 37 °C and 5% CO_2_ by confocal microscopy (SP8, Leica) using a 20×/0.80 NA air immersion objective with excitation using 488-, 577-, and 642-nm laser lines. Images were manually analyzed using ImageJ. Stable NK cell–cancer cell conjugates were defined as a contact occurring over at least two consecutive images (i.e., for longer than 3 min). A kill was defined as an interaction triggering entry of To-pro-3 into the target cell. Contact time was defined as the duration from first contact to time of detachment.

### Treatment with Cytotoxic Proteins/Drugs.

To determine susceptibility to perforin, cells were resuspended in Ca^2+^-HEPES buffer (20 mM HEPES [Sigma], 150 mM NaCl, and 2.5 mM CaCl_2_, pH 7.4) at 1 × 10^7^ cells/mL. An equal volume of native human perforin (Enzo Life Sciences) in 20 mM HEPES buffer with 1% BSA was added and incubated at 37 °C for 15 min unless otherwise described. Cells were resuspended in 200 μL cold PBS with 1 μg/mL PI and analyzed immediately by flow cytometry. Specific lysis was determined relative to baseline lysis without perforin.

To determine susceptibility to SLO (Sigma), SLO was first activated for 10 min in 10 mM dithiothreitol (DTT). SLO was added to cells suspended at 1 × 10^6^/mL in complete RPMI-1640 and incubated at 37 °C for 15 min. Specific lysis determined using PI as previously described. To determine the effect of calcium depletion, cells were instead suspended in PBS containing 2 mM MgCl_2_ or 2 mM CaCl_2_. To test the effect on cholesterol-depleted cells, cells were first treated for 1 h with 5 mM methyl-β-cyclodextrin (Sigma) in complete media at 37 °C.

To determine susceptibility to granzyme B, cells were resuspended at 10^6^/mL in complete RPMI-1640. SLO and granzyme B (Enzo Life Sciences) were then added, and cells were incubated at 37 °C for 4 h. Cell death was quantified as described. Specific death was determined relative to baseline death without SLO and granzyme B.

To determine susceptibility to TRAIL or staurosporine, cells were resuspended in complete RPMI-1640 at 2.5 × 10^5^/mL. Recombinant human TRAIL (R&D Systems) or staurosporine (Sigma) were added, and cells were incubated at 37 °C for 24 h. Specific death was determined as described.

### Calcium Flux.

To measure calcium flux by live confocal microscopy, cells were loaded with 5 µM fluo-4, AM (Invitrogen) for 45 min at 37 °C in complete RPMI-1640 and then washed twice. A total of 3 × 10^4^ cells were allowed to adhere to an 18-well chambered slide (Ibidi) for 30 min. Media were replaced with 10 μL Ca^2+^-HEPES buffer containing 1 μM To-Pro-3, and cells were imaged at 3-s intervals at 37 °C using a 40×/1.1 NA air immersion objective with excitation using 488- and 647-nm laser lines (SP8, Leica). Baseline fluorescence was established over 1 min; then, recombinant human perforin (Abbexa) diluted in Ca^2+^-HEPES buffer was added. To quantify calcium flux, a Gaussian blur was applied to images; then, the mean fluorescence intensity (MFI) of fluo-4 was measured individually for 20 cells per experiment and normalized to the mean baseline fluorescence of each cell.

To measure calcium flux by flow cytometry, cells were loaded with 5 µM indo-1, AM (Invitrogen) for 20 min at 37 °C in complete RMPI-1640. Cells were then washed and resuspended at 1 × 10^5^/mL in RPMI-1640 supplemented with 10% FCS, 1 mM HEPES, 1 mM MgCl_2_, and 1 mM CaCl_2_. Samples were analyzed using flow cytometry with excitation at 405 nm and emission acquired at 450/50 (calcium-free) and 379/28 (calcium-bound). Baseline fluorescence was established for 30 s; then, 1 µg/mL ionomycin or DTT-activated SLO was added to the sample and analysis was continued for 300 s. Indo-1 fluorescence ratio was calculated as the ratio of fluorescence emission at 379/28 to 450/50 nm and normalized to the baseline.

### Perforin Western Blotting.

Western blotting was used to determine perforin content in NK cells and binding of perforin to cancer cells. To determine perforin content in NK cells, lysates were prepared from 5 × 10^5^ treated NK cells using RIPA buffer (50 mM Tris⋅HCl pH 7.4, 1% Nonidet P-40, 388 0.25% Na-deoxycholate, 150 mM NaCl, and 1 mM EDTA in dH_2_O) supplemented with protease inhibitor mixture (Calbiochem) and centrifuged at 18,000 × *g* for 15 min at 4 °C. To measure binding of perforin to cancer cells, 1 × 10^5^ cells were treated with native human perforin as previously described in *Treatment with Cytotoxic Proteins/Drugs*. After 15 min, cells were pelleted at 1,000 × *g* for 5 min at 4 °C and washed in cold PBS. Lysates were prepared in RIPA buffer as described for preparation of NK cell lysates.

To carry out Western blotting, Laemmli buffer containing 10 mM DTT was added to lysates and heated at 95 °C for 10 min. Gel electrophoresis was performed using Bolt 4 to 12% Bis-Tris gels (Invitrogen), and proteins were transferred onto 0.2-μm polyvinylidene fluoride membranes (GE Healthcare), blocked with 5% milk in tris-buffered saline with Tween 20 (10 mM Tris⋅HCl, 15 mM NaCl, and 0.05% Tween 20) for 1 h at RT, and incubated with anti-human perforin antibody (clone: Pf-344; 1 μg/mL; Mabtech) overnight at 4 °C. Membranes were then incubated for 1 h at RT with goat anti-mouse-HRP (1:2,000, Bio-Rad), developed using Clarity Western Enhanced Chemiluminescence Substrate (Bio-Rad), and visualized using a ChemiDoc MP Imager (Bio-Rad).

### Fluorescent SLO Membrane Binding.

To measure binding of SLO, recombinant SLO was conjugated to AF488. Briefly, 10 μg AF488 N-hydroxysuccinimide ester (Themo Fisher) was added to 50 μL 1 mg/mL SLO containing 100 μM NaHCO_3_ and incubated at RT for 1 h. Unbound dye was removed using a Zeba Spin Desalting Column (Thermo Fisher). Cells were treated as previously with DTT-activated AF488-conjugated SLO. After 15 min, cells were washed in cold PBS and centrifuged at 400 × *g* for 5 min at 4 °C. A total of 1 μM To-pro-3 was added to cells, and analysis was performed immediately by flow cytometry.

### Dye Influx Assay.

A total of 3 × 10^4^ Colo829 cells were allowed to adhere to wells of a 0.01% PLL-coated 18-well chambered slide (Ibidi) for 30 min. Media was replaced with 10 µL Ca^2+^-HEPES buffer containing 1 μM To-pro-3 and 200 µM 150-kDa fluorescein isothiocyanate (FITC)-diethylaminoethyl (DEAE)-dextran (Sigma-Aldrich). Cells were imaged at 3-s intervals at 37 °C by confocal microscopy (SP8, Leica) using a 40×/1.1 NA air objective with excitation at 488 nm and 647 nm. Baseline fluorescence was established for 1 min prior to addition of recombinant human perforin diluted in 10 µL Ca^2+^-HEPES buffer containing 1 μM To-pro-3 and 200 µM dextran. Data were analyzed using ImageJ. MFI of To-pro-3 and FITC-dextran was measured over time for 20 cells per experiment and normalized to baseline.

### Surface Phosphatidylserine Detection.

To measure surface phosphatidylserine, 1 × 10^5^ cancer cells were stained with 2 µg/mL annexin V-APC in annexin V binding buffer for 15 min. Cells were washed, and binding of annexin V was assessed by flow cytometry with 1 µg/mL PI used to exclude dead cells.

### Liposome Preparation and Dye Release Assay.

The protocol for liposome preparation and dye release assay was adapted from Hansen et al. (40). Briefly, 1,2-dioleoyl-sn-glycero-3-phosphocholine (Avanti Polar Lipids) and Brain L-α-phosphatidylserine (Avanti Polar Lipids) were dissolved in chloroform and mixed at varying molar concentrations. Lipids were dehydrated and resuspended at 400 µM in liposome buffer (20 mM HEPES and 150 mM Nacl in dH_2_O; pH 7.5) containing 50 mM 5,6-carboxyfluorescein (Sigma-Aldrich) and then sonicated for 2 min. Excess dye was removed using a Nap-5 sephadex column (Cytiva Illustra) followed by three washes by centrifugation at 20,000 × *g* for 10 min and resuspension in buffer.

The 5,6-carboxyfluorescein–containing liposomes were diluted to 20 µM, and 50 µL was added to wells of a 96-well plate. Fluorescence intensity was measured using a plate reader at 1-min intervals (ex: 480 nm, em: 517 nm). Baseline fluorescence was established over 5 min before addition of recombinant perforin. Fluorescence intensity was measured for 60 min followed by addition of 1% Triton X-100 to measure maximal fluorescence from total lysis. To calculate the percentage of fluorescent dye release, 0% was set using baseline fluorescence before perforin addition and 100% was set using maximal fluorescence after Triton X-100 addition.

### In Vivo Killing Assay.

Colo829 cells were treated with 16 Gy either 24 or 72 h prior to inoculation in vivo. The 24- and 72-h-postirradiation cells were labeled with CellTrace CFSE and CTV, respectively, for 15 min at 1 µM. A 1:1 ratio of 24- and 72-h-postirradiation cells (1 × 10^6^ cells each) and 10 × 10^6^ IL-2–cultured human NK cells were injected into the peritoneal cavity of 6- to 8-wk-old female NSG mice (Envigo). At 5 h postinoculation, cells were harvested from the peritoneal cavity by lavage with PBS, and the number of CTV- and CFSE-positive cells was quantified by flow cytometry (NovoCyte; Agilent). The percentage of 72-h-postirradiation cells of total target cells was determined and normalized to the mean percentage in control mice into which no NK cells were injected.

### Statistical Analysis.

Statistical analysis was performed using GraphPad Prism version 8 (GraphPad Software). A Shapiro–Wilk test was used with all data to test for normality. Significant differences were determined as described in figure legends. Statistically significant differences were defined as *P* < 0.05 (*), *P* < 0.01 (**), and *P* < 0.001 (***). Unless otherwise stated, figures show mean ± SD.

## Supplementary Material

Supplementary File

## Data Availability

All study data are included in the article and/or *SI Appendix*.
