# Supplementary material for: Radiotherapy transiently reduces the sensitivity of cancer cells to lymphocyte cytotoxicity
Source: Proc Natl Acad Sci U S A. 2022 Jan 18;119(3):e2111900119. doi: 10.1073/pnas.2111900119 (PMC8785960; doi:10.1073/pnas.2111900119)
Supplement: Supplementary File [file pnas.2111900119.sapp.pdf]

Supplemental Information

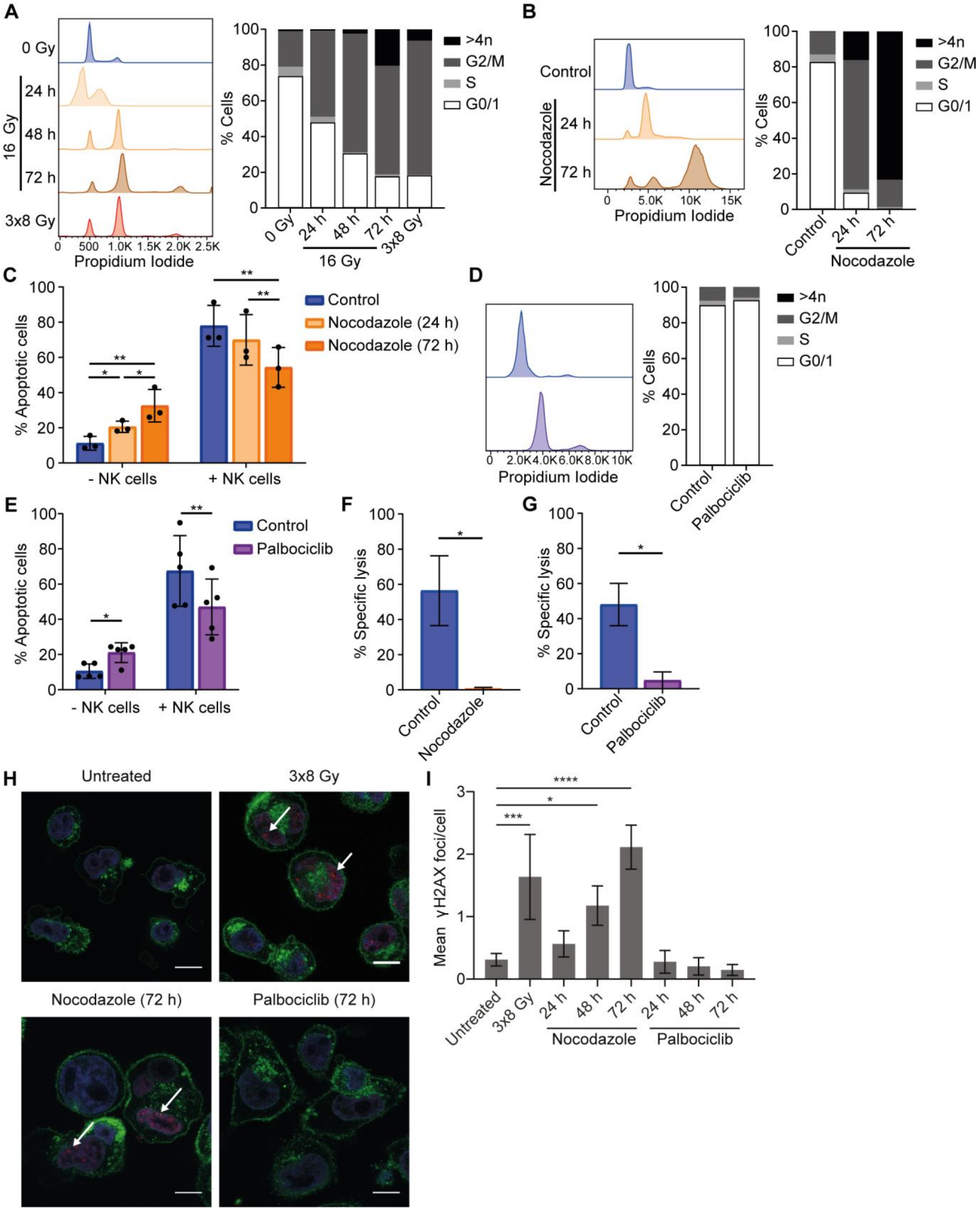

Supplementary Figure 1. Cancer cells in cell cycle arrest are resistant to NK cell cytotoxicity and perforin

(A-B) The cell cycle stage of Colo829 cells was determined using PI 24-72 h after a single 16 Gy dose or 24 h after 3x8 Gy (A) or after 24-72 h treatment with 0.5 µM nocodazole (B). **(C)** Colo829 cells

were treated for 24 or 72 h with 0.5 µM nocodazole and then incubated for 5 h with or without NK cells. Death was determined using annexin V/. **(D-E)** Colo829 cells were treated for 72 h with 5 µM palbociclib and cell cycle stage was assessed by PI (D). Control or palbociclib-treated cells were incubated for 5 h with or without NK cells (E) and lysis was determined as in (C). **(F-G)** Colo829 cells were treated for 72 h with 0.5 µM nocodazole (F) or 5 µM palbociclib (G) and then incubated for 15 min with 500 ng/mL perforin. Specific lysis was determined using PI. **(H-I)** Colo829 cells treated with 3x8 Gy, nocodazole, or palbociclib for indicated times were stained for γH2AX (red; arrows), nuclei (Hoechst 33342; blue), and membrane (WGA; green). Representative images shown in (H). Scale bars indicate 10 µm. Mean γH2AX foci per cell (n=3) shown in (I). Mean ± SD are indicated. Significance was determined using a repeated measures one-way ANOVA (C, I) or paired T-test (E-G). *p<0.05 **p<0.01

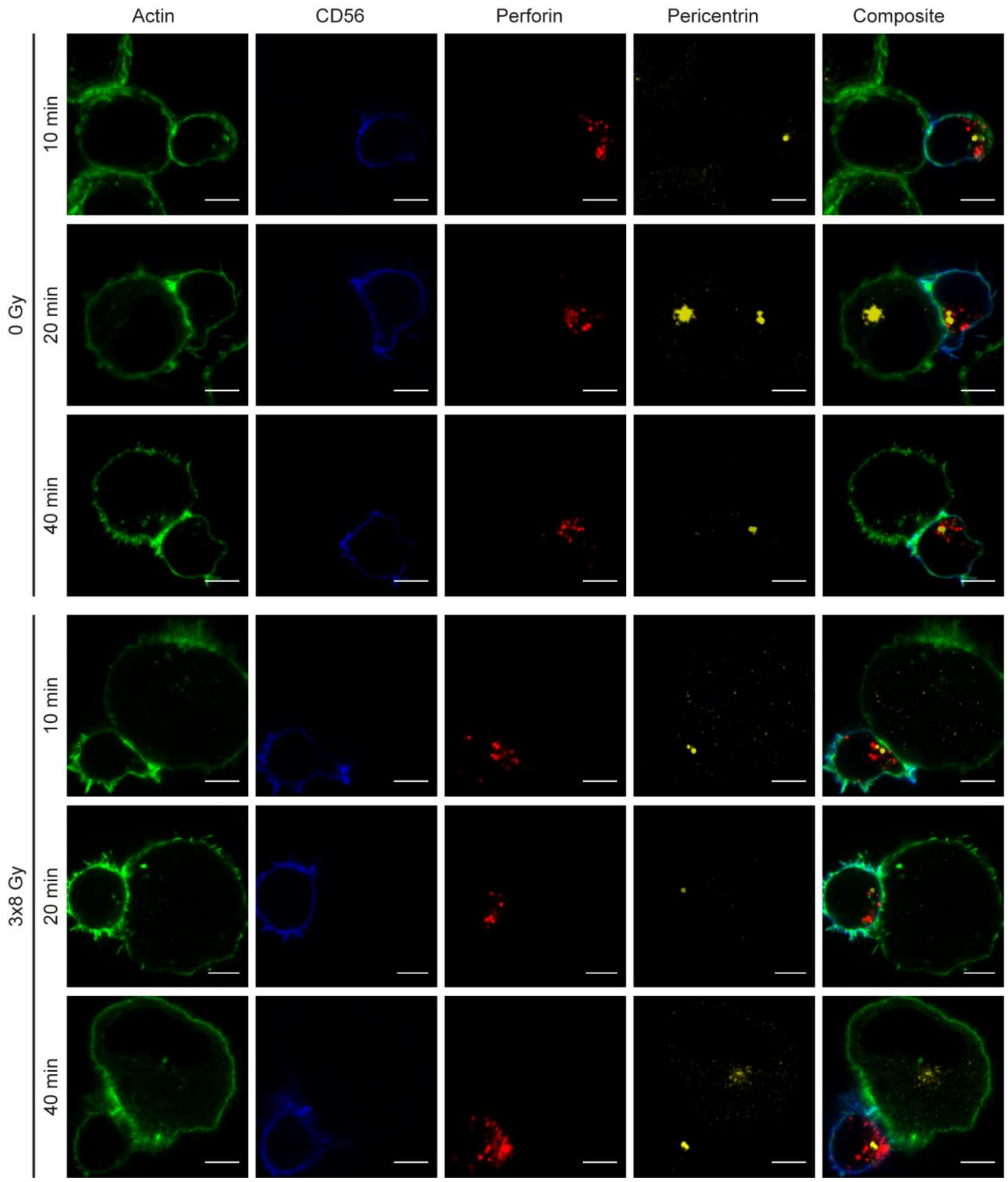

Supplementary Figure 2. NK cell polarization is unaffected by target cell irradiation.

Irradiated or non-irradiated Colo829 cells were incubated with NK cells for 10, 20, or 40 min, then stained for actin (green), CD56 (blue), perforin (red), and pericentrin (yellow), and imaged. Representative images of conjugates are shown for data in Figure 2C-F. Scale bar indicates 5 µm.

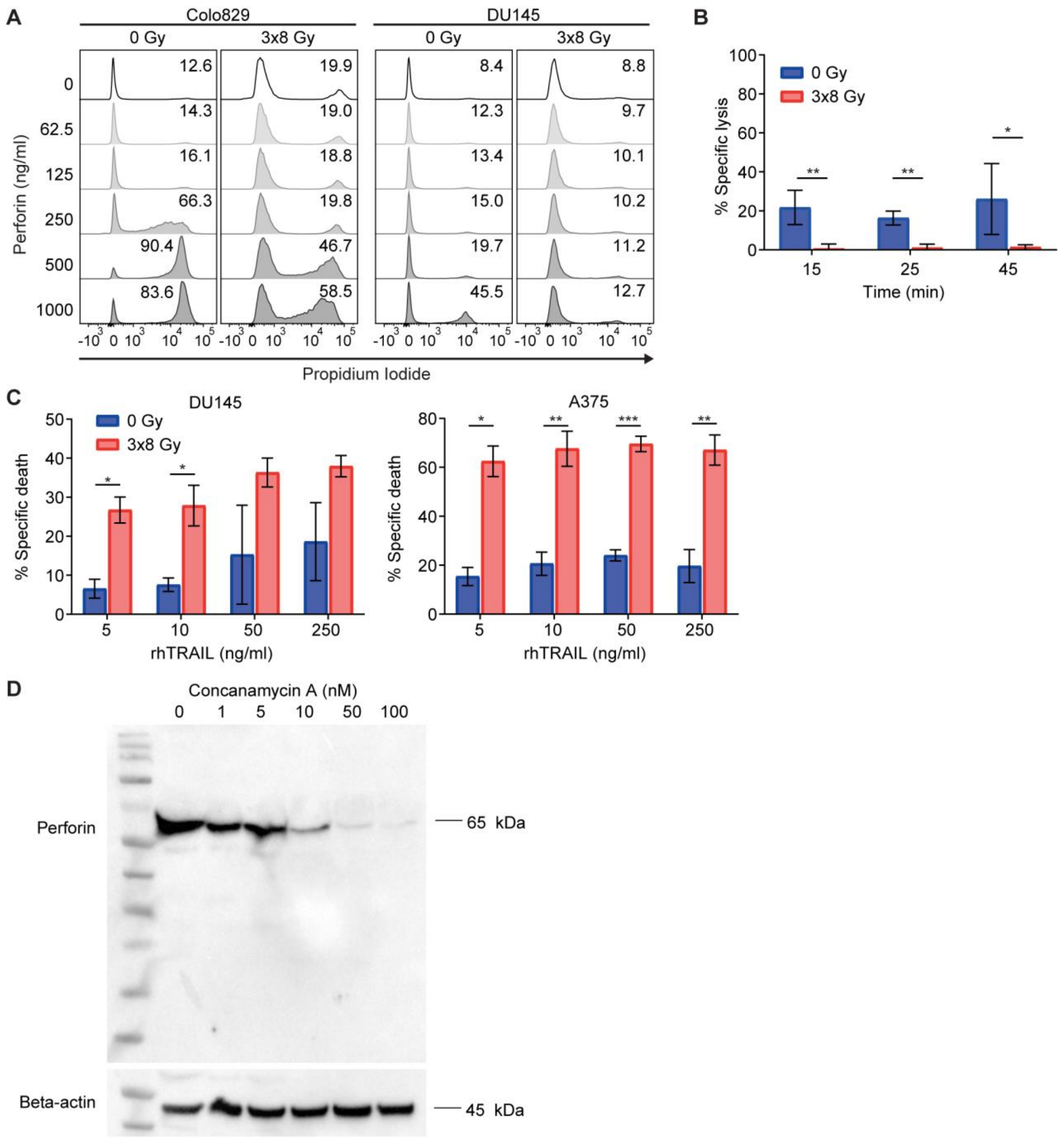

Supplementary Figure 3. Irradiated cancer cells are less susceptible to lysis by perforin.

(A) Irradiated or non-irradiated Colo829 and DU145 cells were treated with native human perforin for 15 min. PI entry was measured using flow cytometry. Representative flow plots and percent of PI+ cells are shown. **(B)** Colo829 cells were treated for 15, 25, or 45 min with 250 ng/mL native human perforin. Specific lysis was assessed using PI. **(C)** DU145 and A375 cells were treated with recombinant human TRAIL (rhTRAIL) for 24 h and specific death was determined by flow cytometry, using annexin V and PI staining. **(D)** Primary human NK cells were treated with varying concentrations of concanamycin A for 2 h. Perforin content was determined by western blotting. β-actin was used as a loading control. Mean ± SD are indicated. Significance was determined using a paired T-test (B-C). $*p<0.05$ $**p<0.01$ $***p<0.001$.

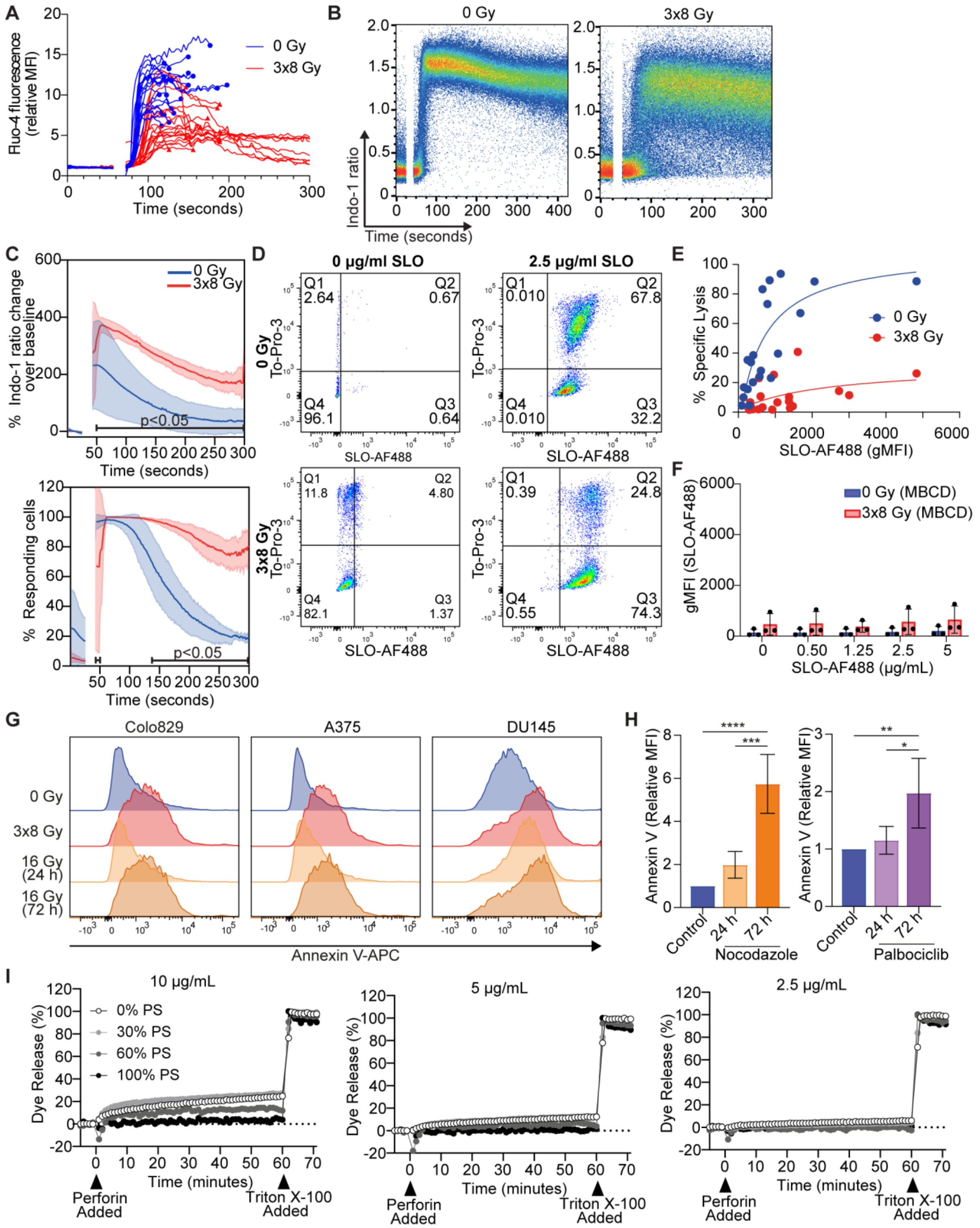

Supplementary Figure 4. Pore formation is inhibited on irradiated cell membranes and correlates with phosphatidylserine.

(A) Irradiated or non-irradiated Colo829 cells were stained with fluo-4-AM and treated with a lytic concentration (40 µg/mL) of recombinant human perforin. Calcium flux was quantified using confocal microscopy for 20 cells per condition by recording the change in fluo-4 fluorescence after perforin addition (at ~60 seconds) relative to baseline. Tracking was stopped upon cell death determined by entry of to-pro-3. Death is indicated by dots at the end of the line trace. **(B)** Colo829 cells were stained with indo-1-AM then treated with 0.5 µg/mL SLO. Calcium flux was assessed using flow cytometry by recording the indo-1 fluorescence ratio after SLO addition (at ~30 sec). Representative flow cytometry plots over time are shown. **(C)** Indo-1 ratio was recorded for irradiated Colo829 cells after addition of 1 µg/mL ionomycin at ~30 sec. The percent indo-1 ratio change over baseline (recorded over initial 26 seconds) and the percent of responding cells were determined (n=3). **(D)** Colo829 cells were treated with AF488-conjugated SLO for 15 minutes and binding was assessed by flow cytometry. Lysis was quantified by entry of To-pro-3. Representative flow cytometry plots are shown. **(E)** Colo829 cells were treated with AF488-conjugated SLO for 15 minutes and binding was assessed by flow cytometry. Lysis was quantified by entry of To-pro-3. A non-linear line of best fit was calculated. **(F)** Cholesterol was depleted from Colo829 cells using methyl-β-cyclodextrin (MBCD) and then cells were treated with SLO-AF488 as in (D) (n=3). Mean ± SD are shown. **(G)** Cancer cells treated with radiotherapy (0 Gy, 3x8 Gy, or 24-72 h after 16 Gy) were stained with annexin V to quantify surface phosphatidylserine. Fluorescence was analyzed by flow cytometry. Representative histograms are shown. **(H)** Colo829 cells treated with cell cycle inhibitors for 24-72 h (nocodazole or palbociclib) were stained with annexin V to quantify surface phosphatidylserine and analyzed by flow cytometry. MFI is shown relative to untreated (control). Significance was determined by one-way ANOVA. Mean ± SD are indicated. **(I)** Liposomes composed of phosphatidylcholine and 0, 30, 60, or 100% phosphatidylserine (PS) and containing 5,6-carboxyfluorescein dye were treated with 10, 5, or 2.5 µg/mL recombinant human perforin. Lysis of liposomes was determined by dye release tracked over time after addition of perforin at 60 s (n=3).

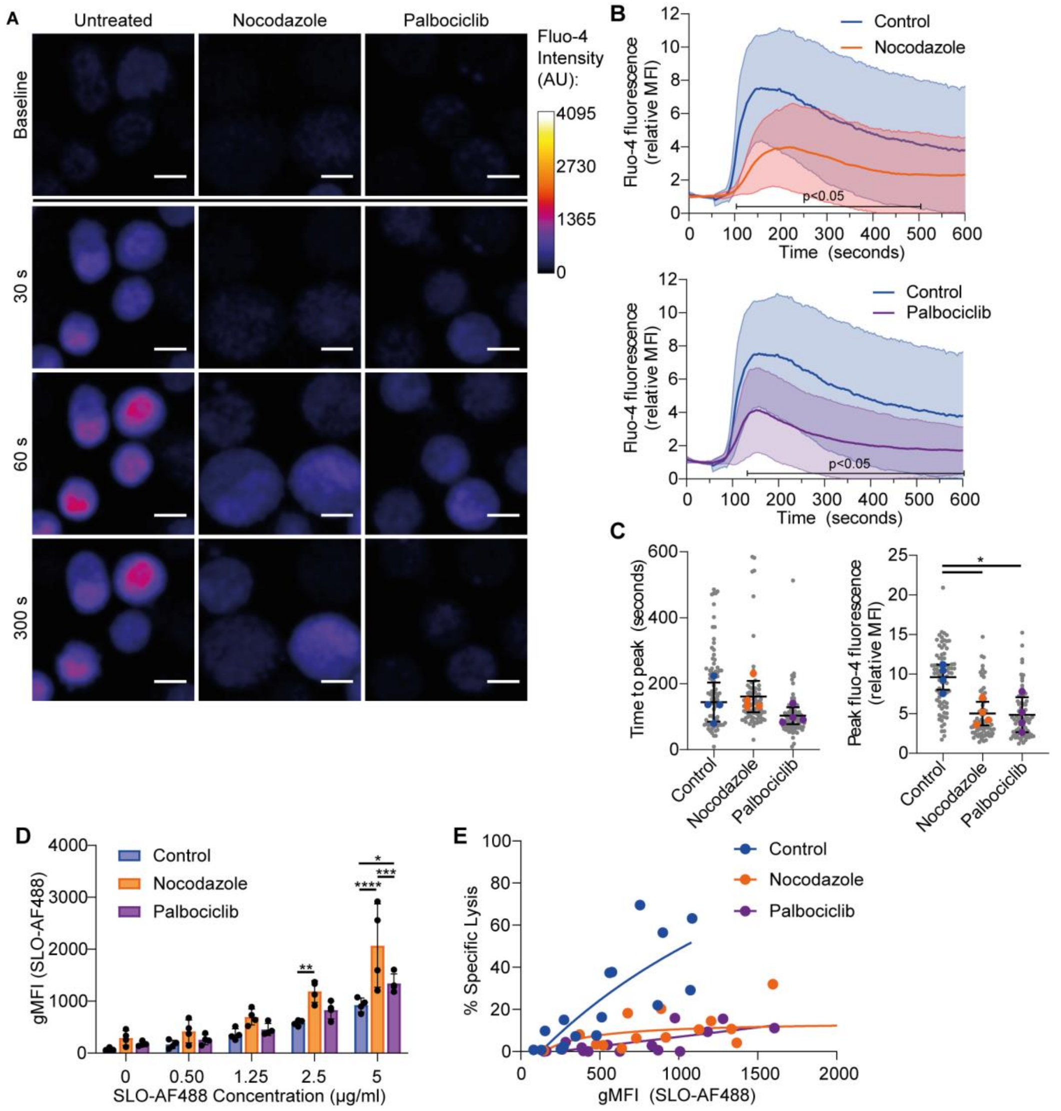

Supplementary Figure 5. Functional pore formation is inhibited on membranes of cells in cell cycle arrest.

(A-C) Colo829 cells were treated for 72 h with nocodazole or palbociclib and stained with fluo-4-AM, then treated with 20 µg/mL recombinant perforin. Calcium flux was quantified using confocal microscopy by recording the change in fluo-4 fluorescence after perforin addition (at ~60 seconds) relative to baseline for 20 cells per condition (n=4). Representative images shown in (A). Scale bars indicate 10 µm. Mean fluo-4 fluorescence relative to baseline over time with perforin added at 60 seconds shown in (B). The peak fluo-4 fluorescence relative to baseline after perforin addition and the time taken to reach peak shown in (C). Grey dots indicate individual cells and coloured dots indicate the mean of each experiment. **(D-E)** Nocodazole- or palbociclib-treated Colo829 cells were treated with AF488-conjugated SLO for 15 minutes and binding was assessed by flow cytometry (D; n=4). Lysis was quantified by entry of To-pro-3 and a non-linear line of best fit was calculated against

SLO-AF488 fluorescence (E). Mean ± SD are indicated. Significance was determined using a one-way ANOVA (C) or two-way ANOVA (B, D). *$p<0.05$ **$p<0.01$ ***$p<0.001$ ****$p<0.0001$.

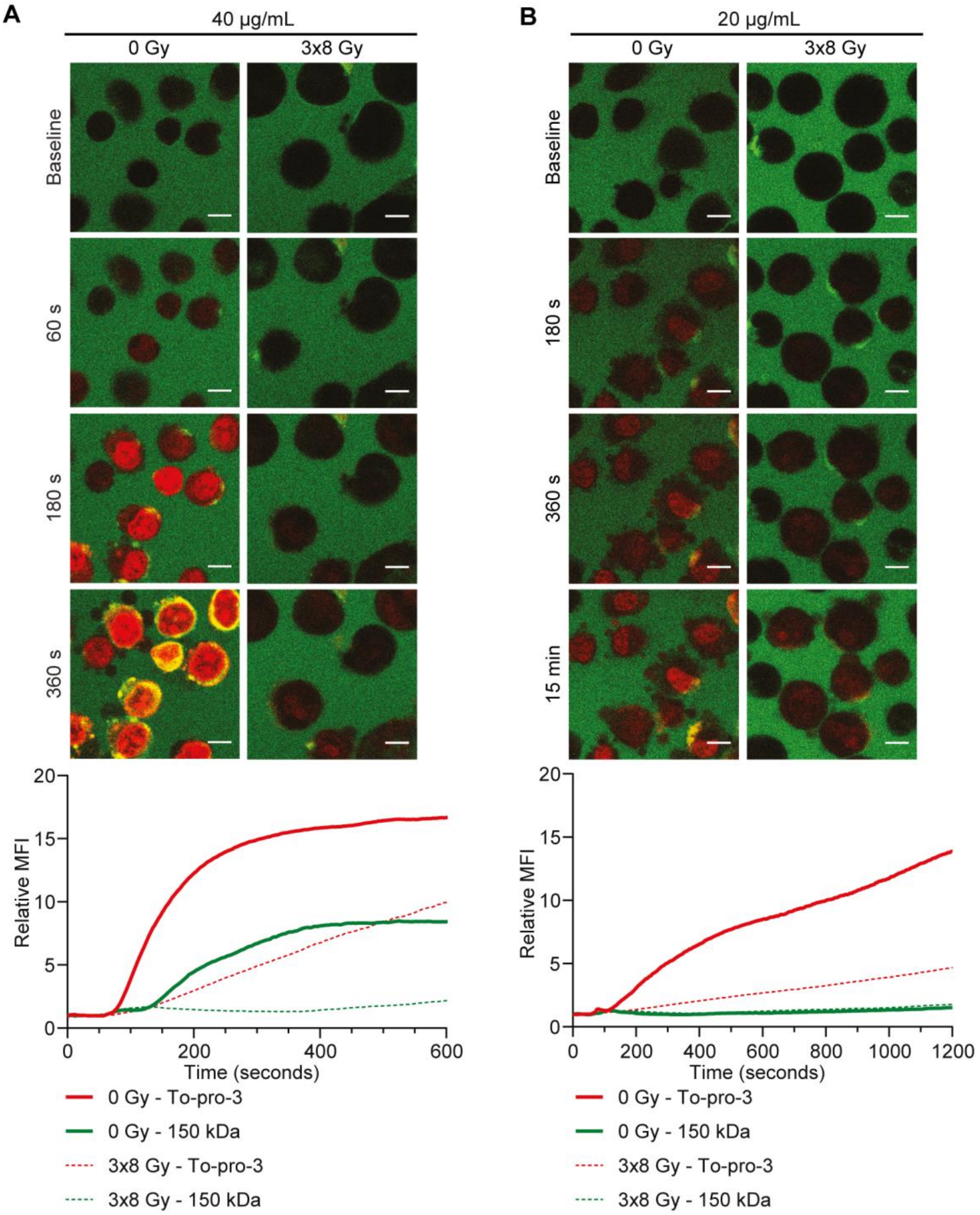

Supplementary Figure 6. Uptake of fluorescent dye through perforin pores is reduced on irradiated cell membranes.

Colo829 cells were treated with 0 Gy or 3x8 Gy and imaged by confocal microscopy at 3 second intervals in the presence of To-pro-3 and 150 kDa FITC-DEAE-dextran. Cells were imaged for 60 seconds to establish baseline fluorescence before addition of 40 μg/mL (A) or 20 μg/mL (B) recombinant human perforin. Representative images of To-pro-3 (red) and dextran (green) fluorescence with 0 Gy and 3x8 Gy-treated Colo829 cells at baseline and at indicated times after perforin addition are shown (top). Entry of To-pro-3 and 150 kDa dextran into cells was quantified by tracking the MFI of individual cells relative to baseline for 20 cells per experiment. The mean of 5 independent experiments is shown. Error bar indicates 10 μm.

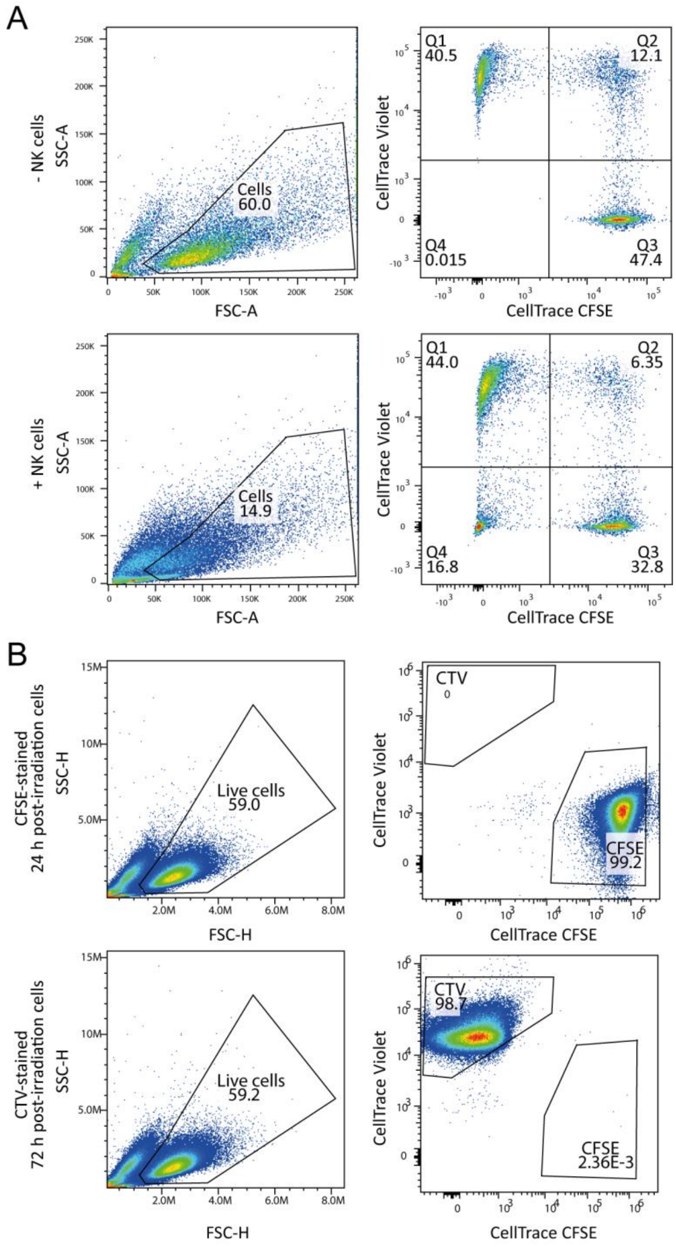

Supplementary Figure 7. Irradiated cancer cells are resistant to NK cell cytotoxicity *in vivo.*

(A) Colo829 cells were treated with 16 Gy either 24 or 72 h prior, stained with CFSE and CTV, respectively, and then mixed at a 1:1 ratio. Targets were then incubated with or without human NK cells at a 5:1 effector-to-target ratio for 5 hours then analyzed by flow cytometry. Results were gated for live cells based on forward and side scatter and the proportion of 24 or 72 h post-irradiation cells was determined by CFSE and CTV staining. Representative flow cytometry plots are shown for data in Figure 6A. **(B)** Representative flow cytometry plot of 72 h and 24 h post-irradiation cells stained with CTV and CFSE, respectively, prior to intraperitoneal injection performed in Figure 6B-D. Gating was performed on live cells based on forward and side scatter and then on CFSE and CTV staining.
